# Supplementary material for: HASTY, the Arabidopsis EXPORTIN5 ortholog, regulates cell‐to‐cell and vascular microRNA movement
Source: EMBO J. 2021 Jun 21;40(15):e107455. doi: 10.15252/embj.2020107455 (PMC8327949; doi:10.15252/embj.2020107455)
Supplement: Supplementary file 2 — Appendix [file EMBJ-40-e107455-s010.pdf]

Appendix for

HASTY, the ArabidopsisEXPORTIN5 ortholog, regulates cell-to-cell and vascular microRNA movement

Florian Brioudes, Florence Jay, Alexis Sarazin, Thomas Grentzinger, Emanuel A. Devers, Olivier Voinnet

Correspondence to: olivier.voinnet@biol.ethz.ch

This file includes:

Appendix Figures S1 to S16
Appendix Table S1

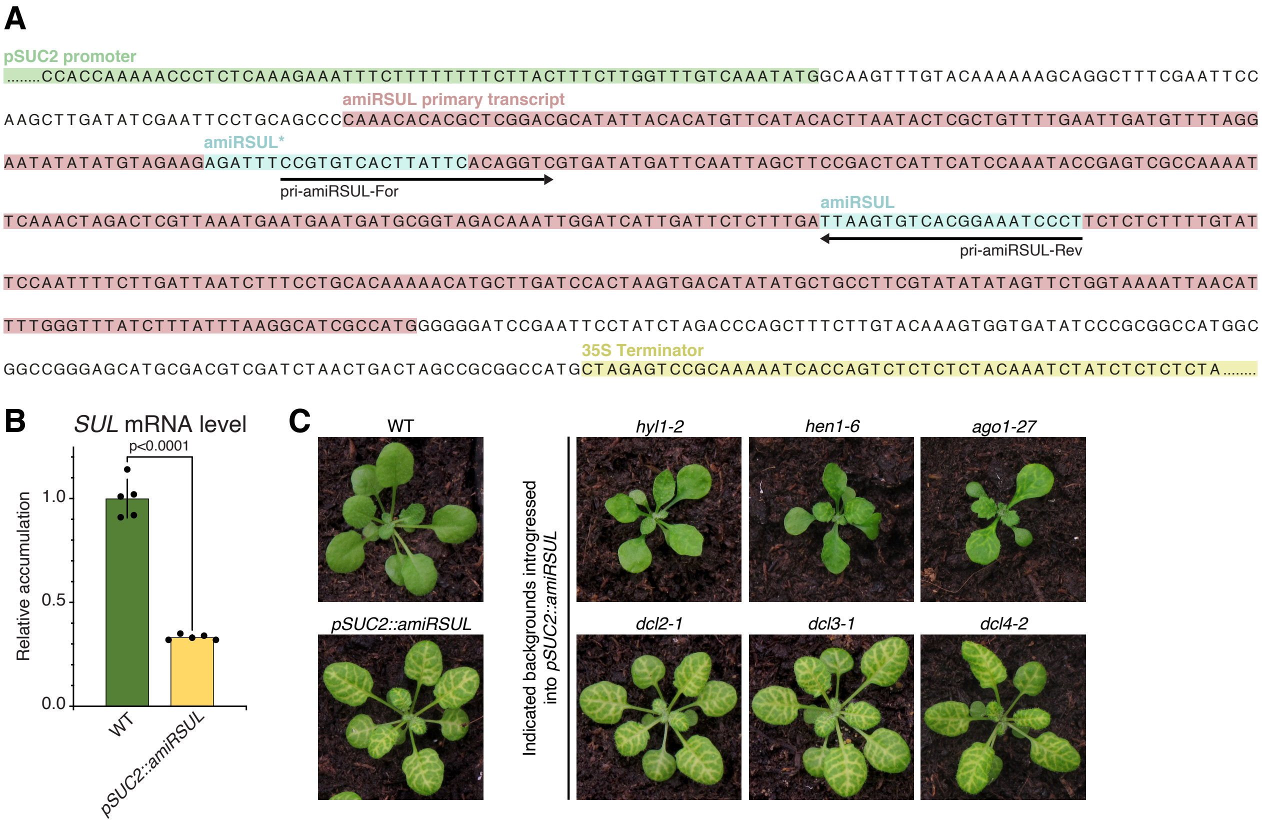

Appendix Figure S1

(A) Scheme of the *pSUC2::amiRSUL* transgene carried by the parental line used in this study. 3'-end of the pSUC2 promoter sequence (green), amiRSUL primary transcript sequence (red), amiRSUL* and amiRSUL mature sequences (blue) and 5'-end of the 35S terminator sequence (yellow) are depicted. Arrows represent pri-amiRSUL-For and pri-amiRSUL-Rev primers used in RT-PCR analyses shown in Fig.S3B.

(B) RT-qPCR analysis of *SUL* mRNA levels in WT and *pSUC2::amiRSUL* leaf. Error-bars: SD. Welch's t-test p-value is indicated. n=5.

(C) Representative phenotype of the *pSUC2::amiRSUL* reporter in the specified genotypes.

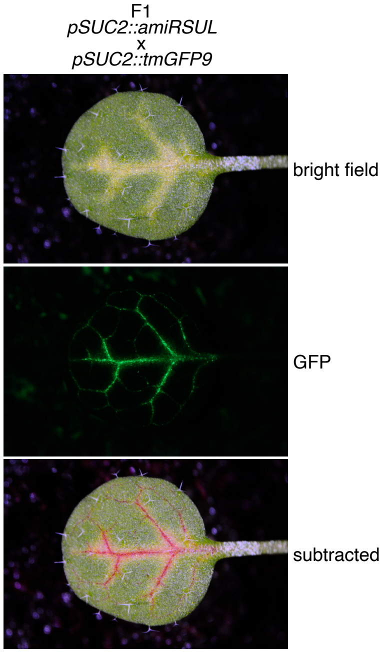

Appendix Figure S2

The *pSUC2::amiRSUL* reporter line was crossed to the *pSUC2::tmGFP9* line expressing a membrane-anchored GFP allele under the control of the *pSUC2* promoter. Plants were imaged for amiRSUL activity manifested as chlorosis (top panel) and GFP expression (middle panel). Subtraction of the GFP channel from the bright field channel (red signal – bottom panel) reveals that the chlorosis extends several cells beyond the GFP signal, which is restricted to the companion cells.

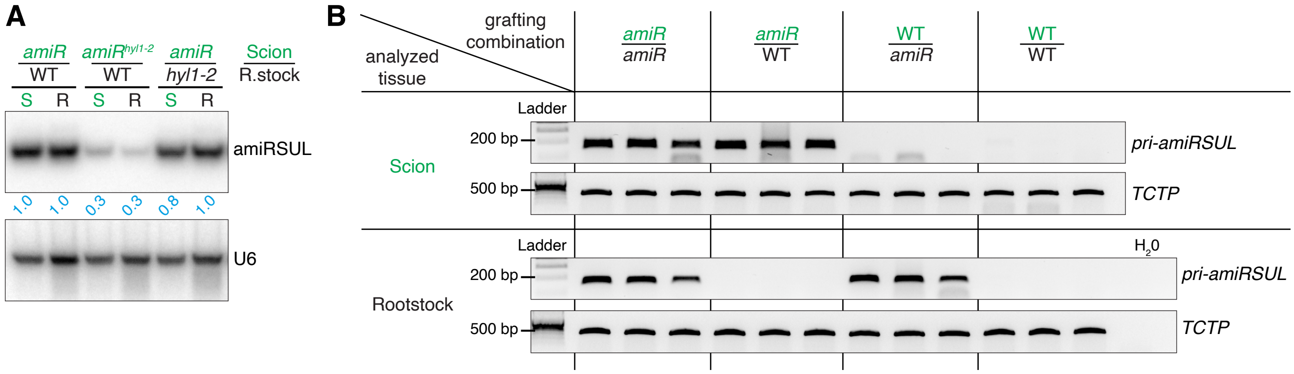

Appendix Figure S3

(A) amiRSUL northern analysis in *pSUC2::amiRSUL* (*amiR*) or *pSUC2::amiRSUL*$^{hyl1\text{-}2}$ scions (S), and in WT or *hyl1-2* rootstocks (R), in the indicated grafting combinations. U6 RNA hybridization provides an internal loading control. Average relative U6-normalized band-intensity quantifications from two biological replicates are indicated.

(B) Agarose gel electrophoresis of RT-PCR products obtained from total RNA extracted from the specified grafted tissues. Primers used for *pri-amiRSUL* amplification are depicted in Figure S1A. Biological triplicates are presented. H_2O was used instead of cDNA as negative control. *TCTP* cDNA amplification was used as positive control for RT-PCR.

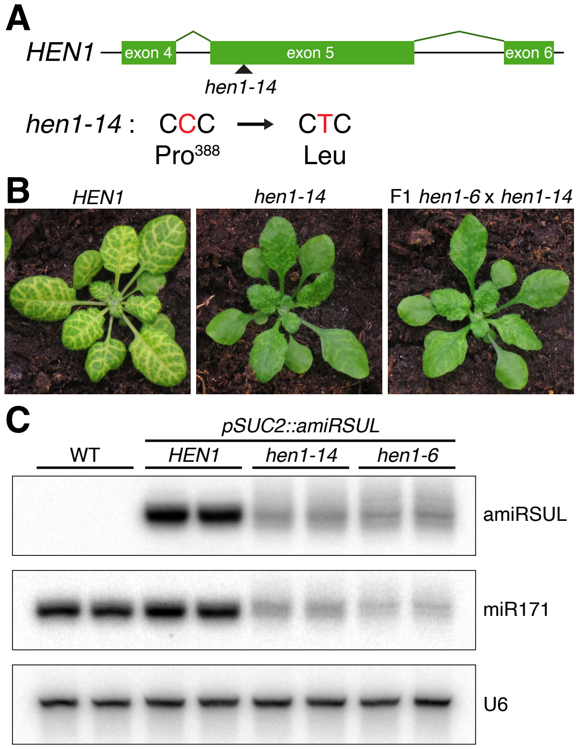

Appendix Figure S4

(A) *HEN1* genomic sequence spanning exons 4-to-6 and position of the *hen1-14* mutation. The EMS-induced nucleotide transition (arrow) in *hen1-14* displayed in red induces a substitution of Proline^{388} into Leucine in the HEN1 protein sequence.

(B) Representative phenotype of the *pSUC2::amiRSUL* reporter line in the *hen1-14* background and in F1 plants resulting from crossing of *hen1-6* with *hen1-14* (allelism test).

(C) Northern analysis (in biological duplicates) of amiRSUL and miR171 accumulation in leaves of the specified genotypes**.** U6 RNA was probed as loading control.

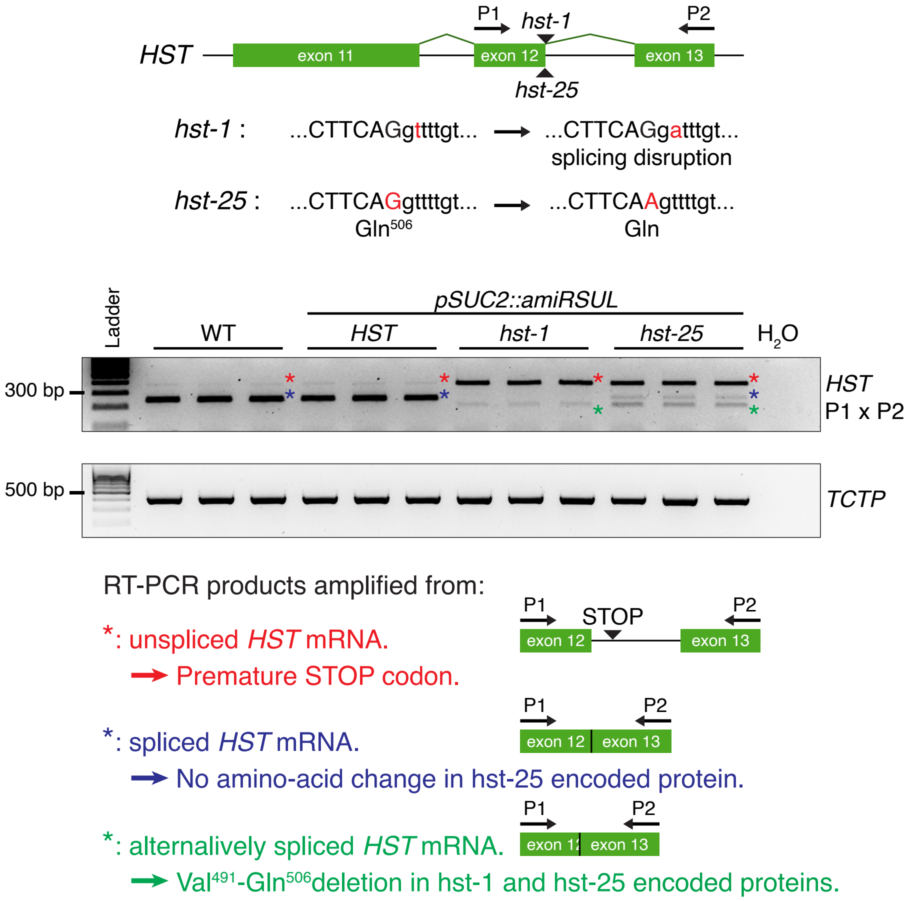

Appendix Figure S5

Top: schematic representation of *HST* genomic sequence positioning *hst-1* and *hst-25* mutations, as well as the P1 and P2 primers used in the bottom RT-PCR experiment. Bottom: agarose gel electrophoresis of RT-PCR products obtained from leaf total RNA of the indicated genotypes. Biological triplicates are presented. H_2O was used instead of cDNA as negative control. P1 and P2 were used to amplify the *HST* sequence surrounding the *hst-1* and *hst-25* mutations. *TCTP* cDNA amplification was used as a positive control for RT-PCR. The *hst-1* and *hst-25* mutations result mainly in a defective splicing, as well as a faint alternative splicing, of intron 12 of *HST* mRNA, as seen with the sequenced RT-PCR products labelled with red and green asterisks, respectively. Normal splicing of intron 12 of *HST* mRNA in the *hst-25* background also occurs in a remnant manner, as detected by the RT-PCR products labelled with blue asterisks.

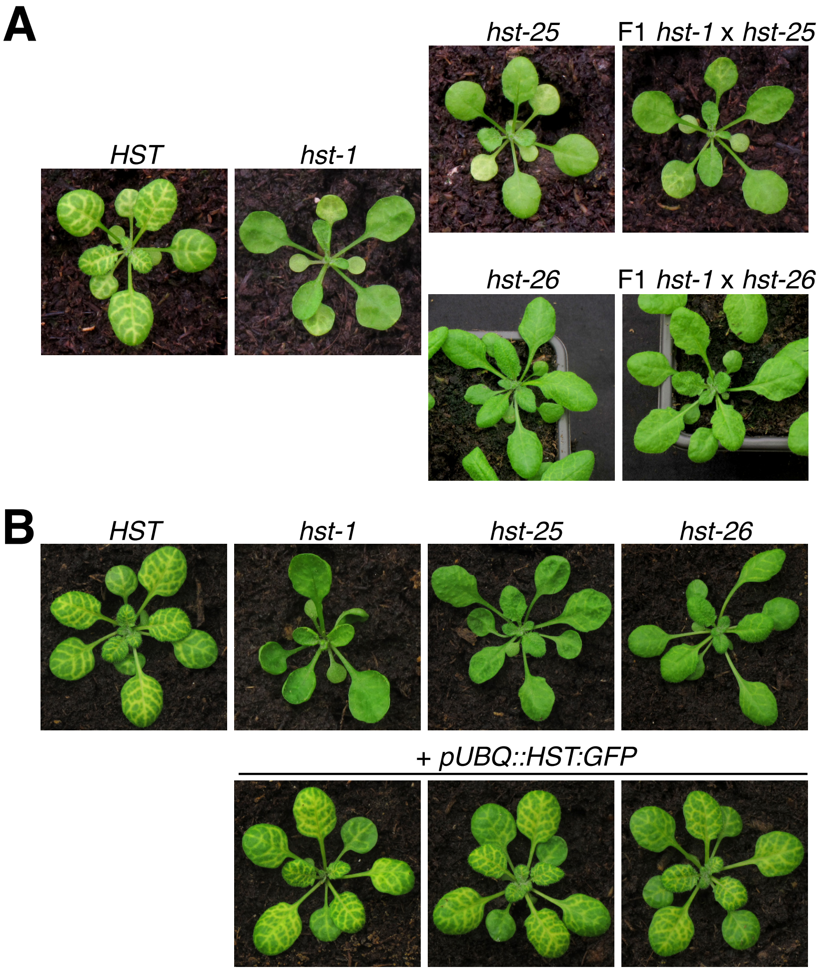

Appendix Figure S6

(A) Representative phenotype of the *pSUC2::amiRSUL* reporter line in the specified genotypes and in F1 plants resulting from crossing of *hst-1* allele with *hst-25* or *hst-26* alleles.

(B) Representative phenotype of the *pSUC2::amiRSUL* reporter line in *hst-1*, *hst-25* and *hst-26* complemented by expressing HST:GFP fusion protein under *UBQ10* promoter (*pUBQ*).

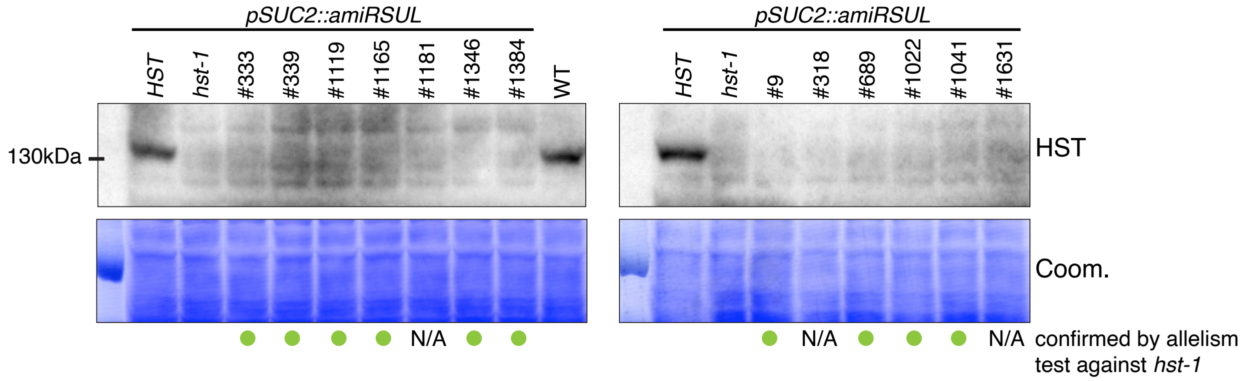

Appendix Figure S7

Western analysis of HST accumulation in leaves of the specified individual mutant plants (indicated by #). WT plants and *pSUC2::amiRSUL* reporter in *HST* or *hst-1* backgrounds were used as controls. Coomassie blue (Coom.) staining of total proteins is shown as a loading control. Allelism test against *hst-1* was conducted and validated for the mutants labelled with green dots. Strong *hst* alleles are fully sterile, precluding such a test in some of the indicated mutants, specified by “N/A”.

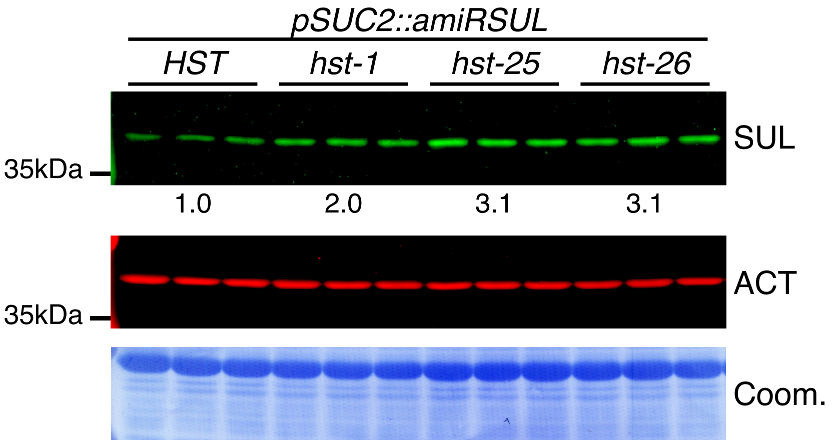

Appendix Figure S8

Western analysis of SULFUR (SUL) and ACTIN (ACT) protein accumulation in the indicated genotypes. Biological triplicates are shown. Coomassie blue (Coom.) staining of total proteins provides a loading control. Average ACT-normalized band relative quantification values are given below the SUL western blot panel.

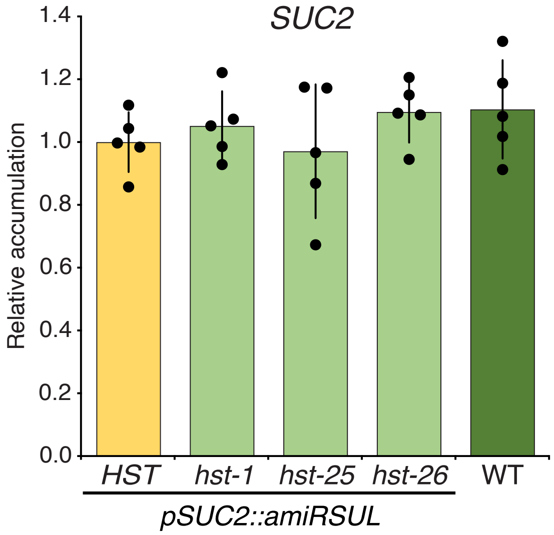

Appendix Figure S9

RT-qPCR-based quantification of the *SUC2* mRNA in the indicated genotypes. Error bars: SD. One-way ANOVA showed no significant difference among the means (p-value=0.5117). n=5.

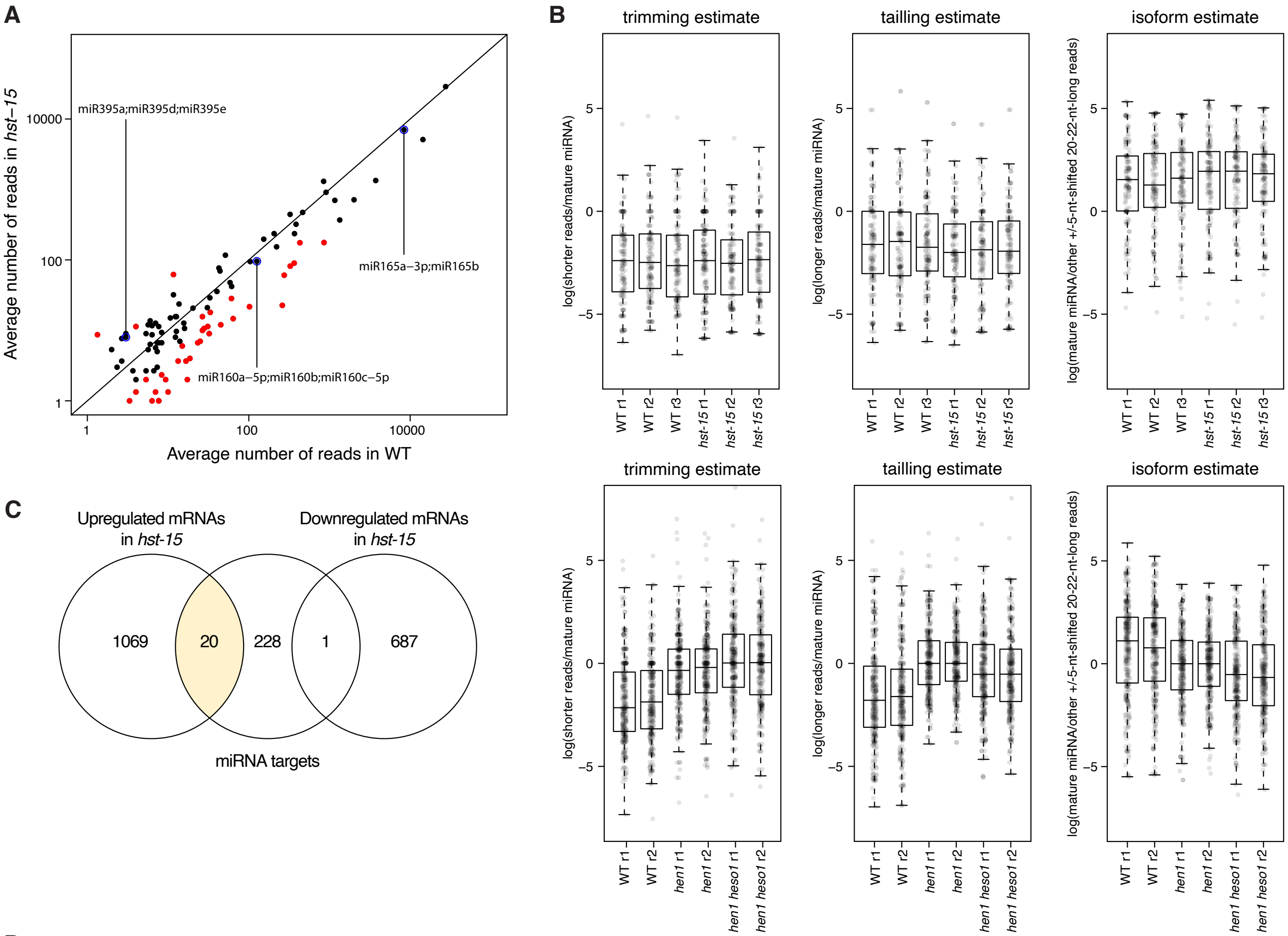

D

mRNA Target id	Target name	Target log2FoldChange	Target padj	Targeting miRNA	Col.0_R1	Col.0_R2	Col.0_R3	hst_R3	hst_R2	hst_R1	miRNA log2FoldChange	miRNA FDR.DE
AT2G33810	SPL3	1.48	0.000	miR156a-5p;miR156b-5p;miR156c-5p;miR156d-5p;miR156e;miR156f-5p	317	518	451	153	234	136	-1.65	0.013
AT1G77850	ARF17	1.07	0.000	miR160a-5p;miR160b;miR160c-5p	120	130	125	79	128	77	-0.77	0.458
AT1G15125	MethylTransferase	0.70	0.000	miR163	269	319	384	399	544	383	0.11	0.536
AT5G53950	CUC2	3.40	0.003	miR164b-3p	3	12	3	0	1	0	-4.73	0.008
AT5G39610	ATNAC6	1.39	0.001									
AT1G30490	PHV	0.58	0.001	miR165a-3p;miR165b and miR166a-3p;miR166b-3p;miR166c;miR166d;miR166e-3p;miR166f;miR166g	7504	7753	9837	6088	8885	6122	-0.60	0.541
AT1G52150	ATHB-15	0.68	0.000									
AT2G34710	PHB	0.80	0.000		24603	26812	31145	24316	35690	26417	-0.28	0.545
AT5G60690	REV	0.53	0.005									
AT5G37020	ARF8	0.48	0.016	miR167a-5p;miR167b	286	372	444	191	305	209	-0.99	0.229
AT1G48410	AGO1	1.23	0.000	miR168a-5p;miR168b-5p	254	383	456	77	117	73	-2.37	0.001
AT3G05690	NF-YA2	1.64	0.000	miR169h;miR169i;miR169j;miR169k;miR169l;miR169m;miR169n	18	35	41	4	8	12	-2.26	0.001
AT1G27340	AT1G27340	0.48	0.004	miR394a;miR394b-5p	27	16	34	10	7	10	-1.81	0.004
AT1G08830	CSD1	0.91	0.000	miR398b-3p;miR398c-3p	1034	1308	1152	545	878	675	-1.09	0.477
AT2G28190	CSD2	1.10	0.000									
AT2G33770	PHO2	1.07	0.000	miR399b;miR399c-3p	42	41	46	32	110	64	0.27	0.471
AT1G31280	AGO2	1.39	0.000	miR403-3p	1230	1387	1397	328	438	336	-2.22	0.055
AT1G12220	RPS5	0.62	0.023	miR472-3p	21	26	25	3	10	5	-2.40	0.001
AT5G63020	AT5G63020	0.38	0.036									
AT1G66230	MYB20	1.08	0.000	miR858a	5	3	5	8	10	6	0.52	0.445

Appendix Figure S10

(A) Scatter plot representation of miRNA abundance in *hst-15* vs. WT. Significantly differentially expressed miRNAs in *hst-15* are highlighted in red. The various forms of miR160, miR165/166 and miR395, of which the movement is studied in Fig.7, are indicated.
(B) Trimming, tailing and isoform estimates of miRNA reads in *hst-15* vs. WT (top) and in *hen1*, *hen1 heso1* vs. WT (bottom).
(C) Venn diagram showing the overlaps of significantly upregulated and downregulated transcripts in *hst-15* with 249 validated/predicted miRNA target transcripts of *Arabidopsis*.
(D) List of miRNA targets upregulated in *hst-15* obtained in (C), crossed with the results of differential analysis of sRNA deep sequencing data in *hst-15*. Significantly differentially expressed miRNAs in *hst-15* are highlighted in red. Details of sRNA sequencing data and bioinformatics methods used can be found in the material and methods section.

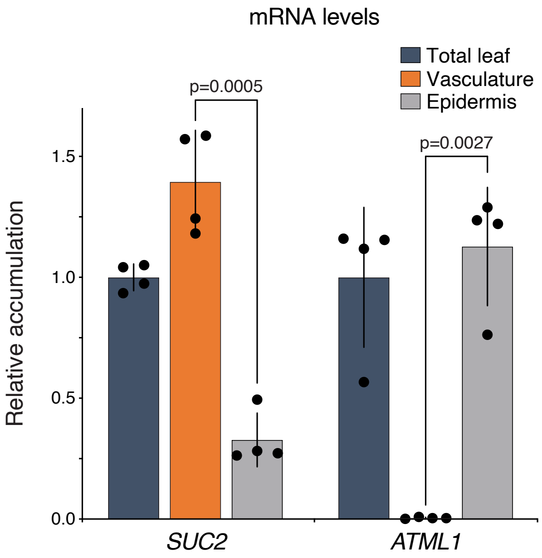

Appendix Figure S11

RT-qPCR-based quantification of *SUC2* and *ATML1* mRNA levels in total, vasculature and epidermis leaf tissues validating the Meselect procedure used in Fig.4A-B and Fig.S12A. Bars represent the mean enrichment in tissues of the four genetic backgrounds used. Error bars: SD. Welch's t-test p-values are indicated. n=4.

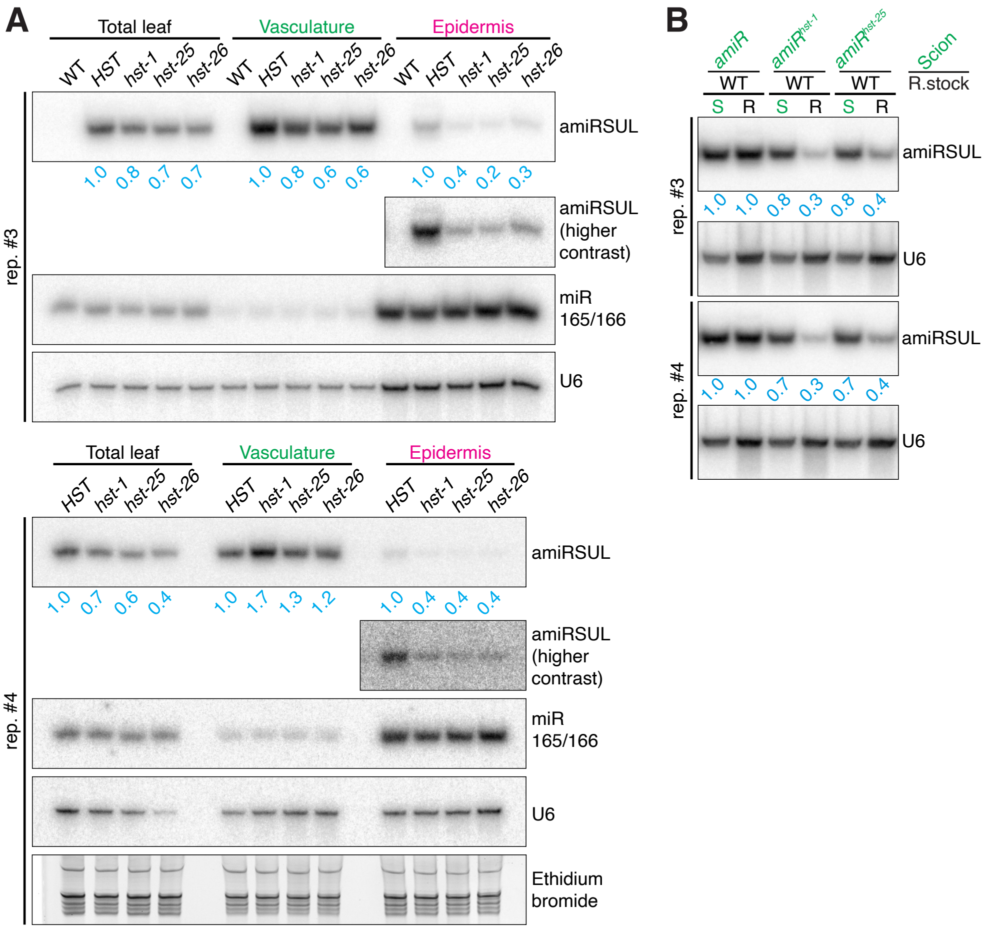

Appendix Figure S12

(A) Independent biological replicates (rep.) of amiRSUL northern analyses shown in Fig.4A, in vasculature and epidermis tissues separated with the Meselect procedure, in the indicated genotypes. An enhanced contrast is shown for the epidermis lanes. miR165/166 and U6 were probed as endogenous controls. Ethidium bromide staining of 5S rRNA and tRNA is shown as loading control in rep.#4. For each tissue, relative amiRSUL band-intensity quantifications normalized with U6 signals (rep.#3) or rRNA signals (rep.#4, in which U6 transfer appeared to be incomplete) are indicated.

(B) Independent biological replicates of amiRSUL northern analyses shown in Fig.4C, in *pSUC2::amiRSUL* (*amiR*), *amiR*$^{hst\text{-}1}$ and *amiR*$^{hst\text{-}25}$ scions (S) and WT rootstocks (R), in the indicated grafting conditions. U6: as in (A). Relative U6-normalized band-intensity quantifications are indicated for each tissue.

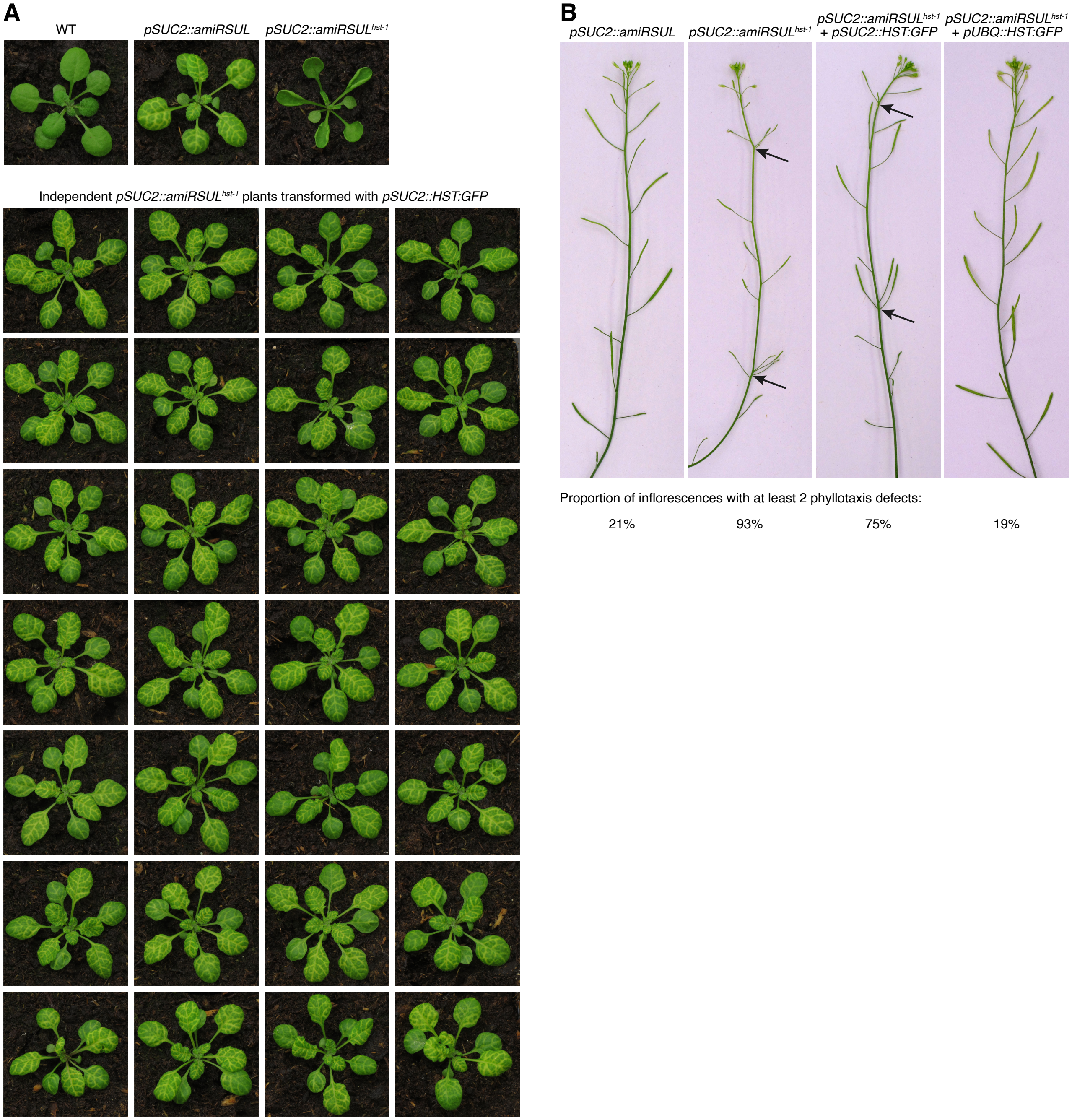

Appendix Figure S13

(A) Genetic mosaic experiment: 28 independent *pSUC2::amiRSUL*$^{hst\text{-}1}$ primary transformants expressing *pSUC2::HST:GFP* are shown and compared to WT, *pSUC2::amiRSUL* and *pSUC2::amiRSUL*$^{hst\text{-}1}$ plants grown side-by-side.

(B) Representative phenotype of *pSUC2::amiRSUL* inflorescences in the indicated genetic backgrounds. Arrows indicate phyllotaxis pattern defects. Proportion of inflorescences with at least 2 phyllotaxis defects is presented at the bottom. $n \geq 42$.

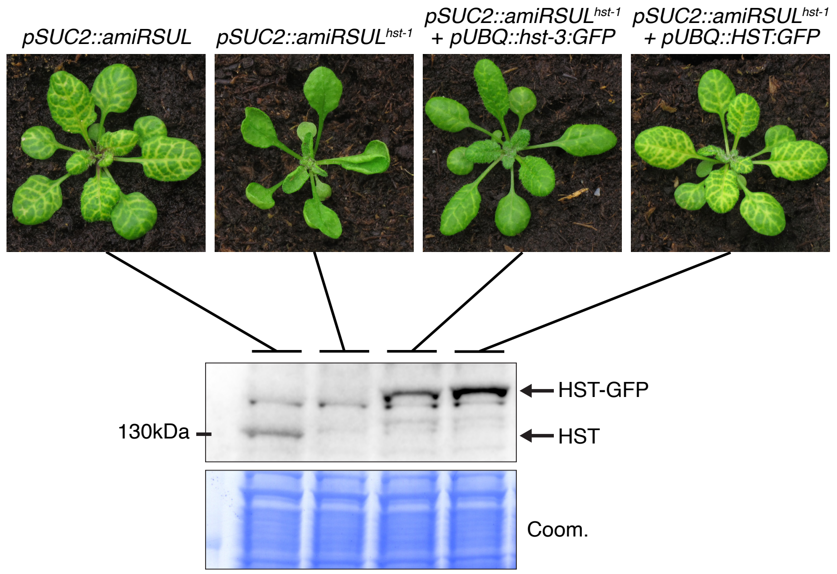

Appendix Figure S14

Representative phenotype of the *pSUC2::amiRSUL* reporter in plants expressing hst-3-GFP under *pUBQ*. *pSUC2::amiRSUL* in WT and *hst-1* backgrounds, as well as *pSUC2::amiRSUL*$^{hst\text{-}1}$ expressing HST-GFP under *pUBQ* are shown as controls. HST protein detection by western analysis in leaves of the corresponding plants is presented (bottom). The bands corresponding to HST and the HST-GFP fusion protein are indicated by arrows. Coomassie blue (Coom.) staining of the western blot membrane provides a loading control.

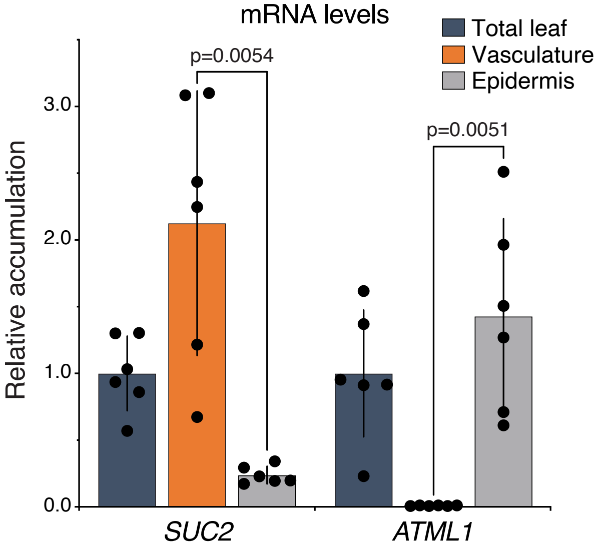

Appendix Figure S15

RT-qPCR-based quantification of *SUC2* and *ATML1* mRNA levels in total, vasculature and epidermis leaf tissues validating the Meselect procedure used in Fig.7C-D. Bars represent the mean enrichment in tissues of the two genetic backgrounds used. Error bars: SD. Welch's t-test p-values are indicated. n=6.

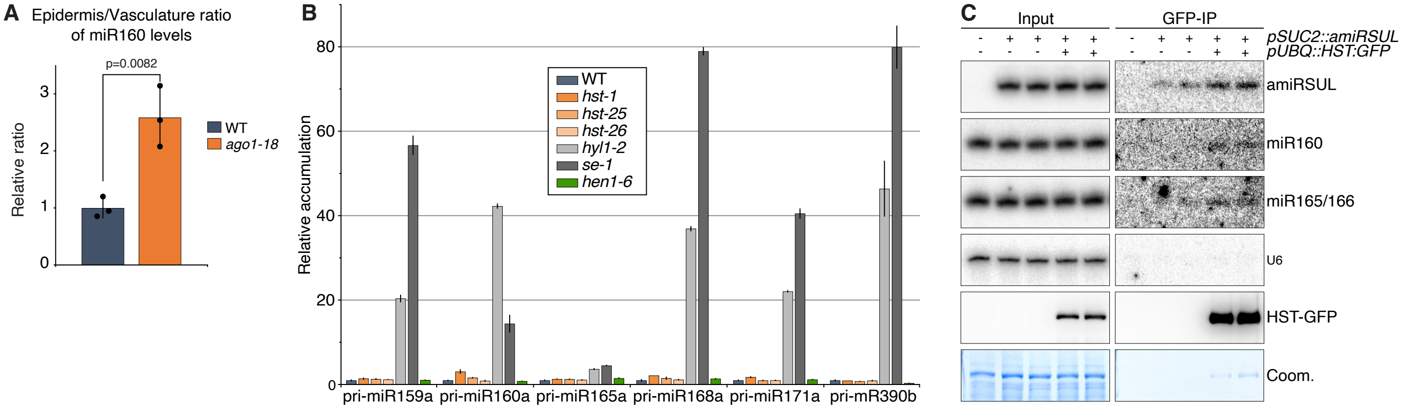

Appendix Figure S16

(A) Epidermis/vasculature ratio of miR160 levels measured by stem-loop RT-qPCR in leaf of WT and *ago1-18* mutant plants. Error bars: SD. t-test p-value is indicated. n=3.

(B) RT-qPCR analyses of the indicated primary miRNA transcript accumulation in leaves of the specified WT or mutant backgrounds. Error bars: SD. n=2.

(C) Northern analysis of amiRSUL, miR160 and miR165/166 in input and GFP-IP fractions from leaves of WT plants, *pSUC2::amiRSUL* plants (in duplicates) and *pSUC2::amiRSUL*$^{hst\text{-}1}$ plants complemented with *pUBQ::HST:GFP* (in duplicates). U6 serves as a loading control. Note the non-specific binding of amiRSUL and miR165/166 in the GFP-IP fraction of the *pSUC2::amiRSUL* control condition. HST-GFP detection by western analysis and Coomassie blue (Coom.) staining of the western blot membrane are provided.

Appendix Table S1

Sequences of primers used this study.

Application	Primer name	Sequence	AGI locus identifier of amplified sequence
Cloning	pSUC2-attB4-For	GGGGACAACTTTGTATAGAAAAGTTGCTGCATGCAAAATAGCACACCAT	At1g22710
	pSUC2-attB1r-Rev	GGGGACTGCTTTTTTGTACAAACTTGCCATATTTGACAAACCAAGAAAG	
	pUBQ10-attB4-For	GGGGACAACTTTGTATAGAAAAGTTGCTAGTCTAGCTCAACAGAGCTTTTA	At4g05320
	pUBQ10-attB1r-Rev	GGGGACTGCTTTTTTGTACAAACTTGCCTGTTAATCAGAAAAACTCAGAT	
	HST-attB1-For	GGGGACAAGTTTGTACAAAAAAGCAGGCTATGGAAGATAGCAACTCCACG	At3g05040
	HST-attB2-Rev	GGGGACCACTTTGTACAAGAAAGCTGGGTCTTGTACGAACTCTTCATCCCAC	
	hst-3-mut-For	CAATTCCTGGCTGTAAAATCTGGAGATGTC	At3g05040
	hst-3-mut-Rev	GATTTTACAGCCAGGAATTGGACCGCCGAT	
RT-PCR	pri-amiRSUL-For	CCGTGTCACTTATTCACAGGTC	
	pri-amiRSUL-Rev	AGGGATTTCCGTGACACTTAA	
	HST-P1	CTAGAATTGATCAAGCTTACTGC	At3g05040
	HST-P2	CCTCAAATATTCCACGTAATGC	
	TCTP-For	GAGCTTCTGTCTGACTCTTTCCC	At3g16640
	TCTP-Rev	GTTGAACCCTCCTTGTAGTAAGC	
RT-qPCR	SUL-qFor	GCAGGACAAGCTTCAAGACC	At4g18480
	SUL-qRev	CGGTGCCTTAAGCAGTTAGG	
	SUC2-qFor	TAGCCATTGTCGTCCCTCA	At1g22710
	SUC2-qRev	CCTAACACAAATGCTGGAATGT	
	ATML1-qFor	GAGGAGGAGGAGGTAGTGCT	At4g21750
	ATML1-qRev	TGTGAGTAGTGAACCGCCAC	
	APS4-qFor	GCTGGGCAAGTCCTCTCAAA	At5g43780
	APS4-qRev	CGGAACCGACATGTTGACGA	
	pri-miR159a-qFor	GGTCTTTACAGTTTGCTTATG	At1g73687
	pri-miR159a-qRev	AGAAGGTGAAAGAAGATGTAG	
	pri-miR160a-qFor	TGCCTGGCTCCCTGTATG	At2g39175
	pri-miR160a-qRev	CCATCCACGGAGGTCATC	
	pri-miR165a-qFor	GATCGATTATCATGAGGGTTAAGC	At1g01183
	pri-miR165a-qRev	CTATAATATCCTCGATCCAGACACC	
	pri-miR166a-qFor	GGGACGAACATAGAAAGAGAGAGA	At2g46685
	pri-miR166a-qRev	AATATGGAGTAAACAGGGAGCAAC	
	pri-miR168a-qFor	ATAAACCTCATTTCCCCATTACAA	At4g19395
	pri-miR168a-qRev	TTCCAGATCTGATAGGATTTACGA	
	pri-miR171a-qFor	TGATATTGGCCTGGTTCACTCA	At3g51375
	pri-miR171a-qRev	CGCGGCTCAATCAATCAGATAATCT	
	pri-miR390b-qFor	TGTAAAGGAAGATGCATACCTATGGA	At5g58465
	pri-miR390b-qRev	AAGGAGGGAATGAAGTAGGAATCACATA	
	SHR-qFor	TTGATCTTGTGGCTTGTGAGC	At4g37650
	SHR-qRev	TACCATCGACCAAACACCTTC	
	SCR-qFor	CTGGCTCTGATGCACACACTC	At3g54220
	SCR-qRev	ACTCTCTTCGCCGTAGCTTGC	
	PHB-qFor	CTTTCCAAGGCTACAGGAACTG	At2g34710
	PHB-qRev	AGCAACCTTCATGGTTCTAAA	
	PHV-qFor	CGTGATGTTAACAACCCAGCTA	At1g30490
	PHV-qRev	CGTGAAACAGCTACGATACCAA	
	ARF17-qFor	AGCACCTGATCCAAGTCCTTCTATG	At1g77850
	ARF17-qRev	TGGTGAATAGCTGGGGAGGATTTC	
	ACT2-qFor	GCACCCTGTTCTTCTTACCG	At3g18780
	ACT2-qRev	AACCCTCGTAGATTGGCACA	
	RHIP1-qFor	GAGCTGAAGTGGCTTCAATGAC	At4g26410
	RHIP1-qRev	GGTCCGACATACCCATGATCC	
	YLS8-qFor	GAGTCTAGTGATTCTGGTCAG	At5g08290
	YLS8-qRev	CACTGAATCATGTTCGAAGCAAGT	
pSUC2::amiRSUL genotyping primers	pSUC-amiRSUL-genomic-For	GTAGTCAACTGCTGTAGATGTC	Between At3g19660 and At3g19663
	pSUC-amiRSUL-genomic-Rev	TATTTTCCAGAGCAGATATCGG	
	pSUC-amiRSUL-TDNA-LeftBorder	CAAAATATAGCGCGCAAACTAG	
Probes for small RNA northern blot	amiRSUL	AGGGATTTCCGTGACACTTAA	
	amiRSUL*	GAATAAGTGACACGGAAATCT	
	miR159	TAGAGCTCCCTTCAATCCAAA	
	miR160	TGGCATACAGGGAGCCAGGCA	
	miR160*	CATGCTTGACTCCTTGTACGC	
	miR165/166	GGGGGATGAAGCCTGGTCCGA	
	miR171	GATATTGGCGCGGCTCAATCA	
	miR390	GGCGCTATCCCTCCTGAGCTT	
	miR390*	TGAAACTCAGGATGGATAGCG	
	miR395	GAGTTCCCCCAAACACTTCAG	
	miR822	CATGTGCAAATGCTTCCCGCA	
	miR822*	CCTGTAGAAAGCATTTGCACA	
	U6	AGGGGCCATGCTAATCTTCTC	
Stem-loop RT-qPCR	miR160-RT	GTCGTATCCAGTGCAGGGTCCGAGGTATTCGCACTGGATACGACTGGCAT	
	miR395a-RT	GTCGTATCCAGTGCAGGGTCCGAGGTATTCGCACTGGATACGACGAGTTC	
	snoR85-RT	GTCGTATCCAGTGCAGGGTCCGAGGTATTCGCACTGGATACGACACATGT	
	U6-RT	GTCGTATCCAGTGCAGGGTCCGAGGTATTCGCACTGGATACGACAAAATTTG	
	miR160-qFor	CCGGCGTGCCTGGCTCCCTGT	
	miR395a-qFor	CCGGCGCTGAAGTGTTTGGGG	
	snoR85-qFor	CCGGCGGTGCATTCAAAAGCCCTT	
	U6-qFor	CACGCATAAATCGAGAAATGGTC	
	Universal Rev	AGTGCAGGGTCCGAGGTATTC	
