# Supplementary figures and images for: HASTY, the Arabidopsis EXPORTIN5 ortholog, regulates cell‐to‐cell and vascular microRNA movement
Source: EMBO J. 2021 Jun 21;40(15):e107455. doi: 10.15252/embj.2020107455 (PMC8327949; doi:10.15252/embj.2020107455)

Appendix Figure S6A

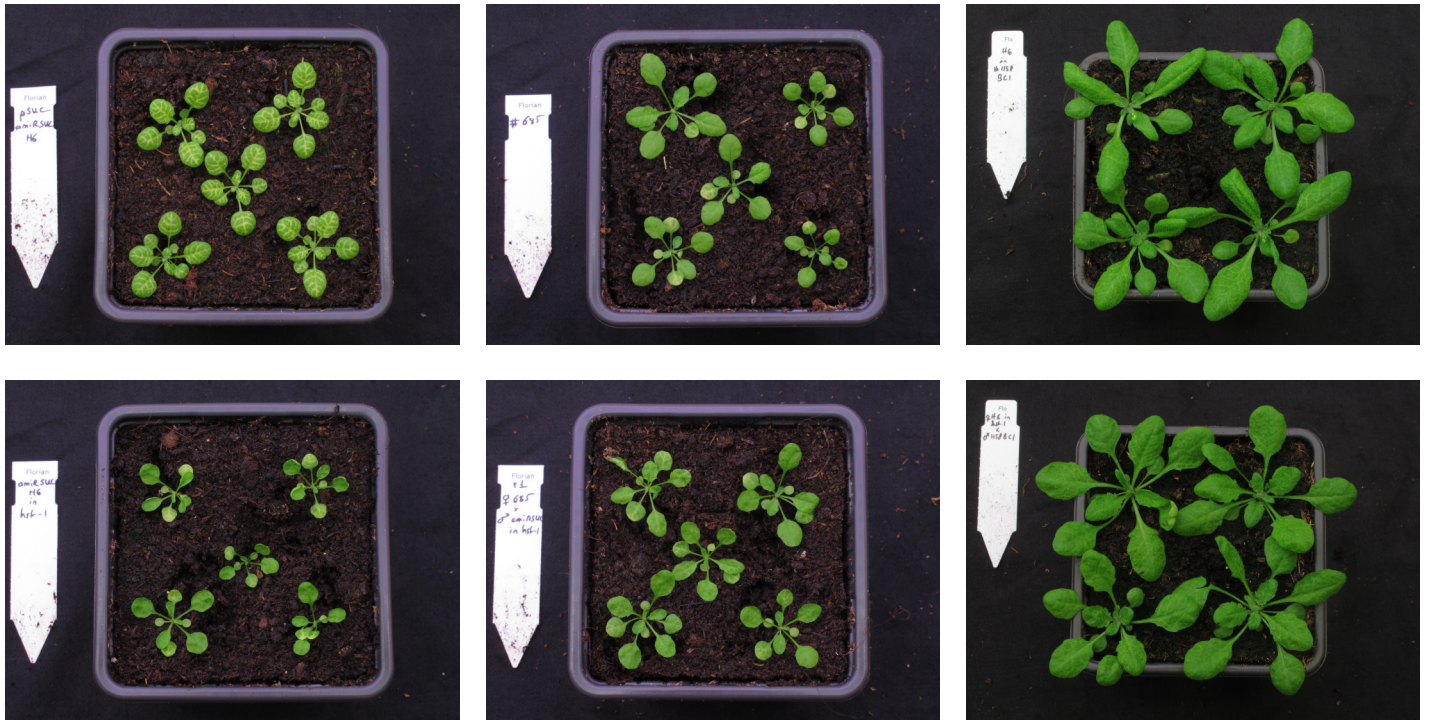

Appendix Figure S6B

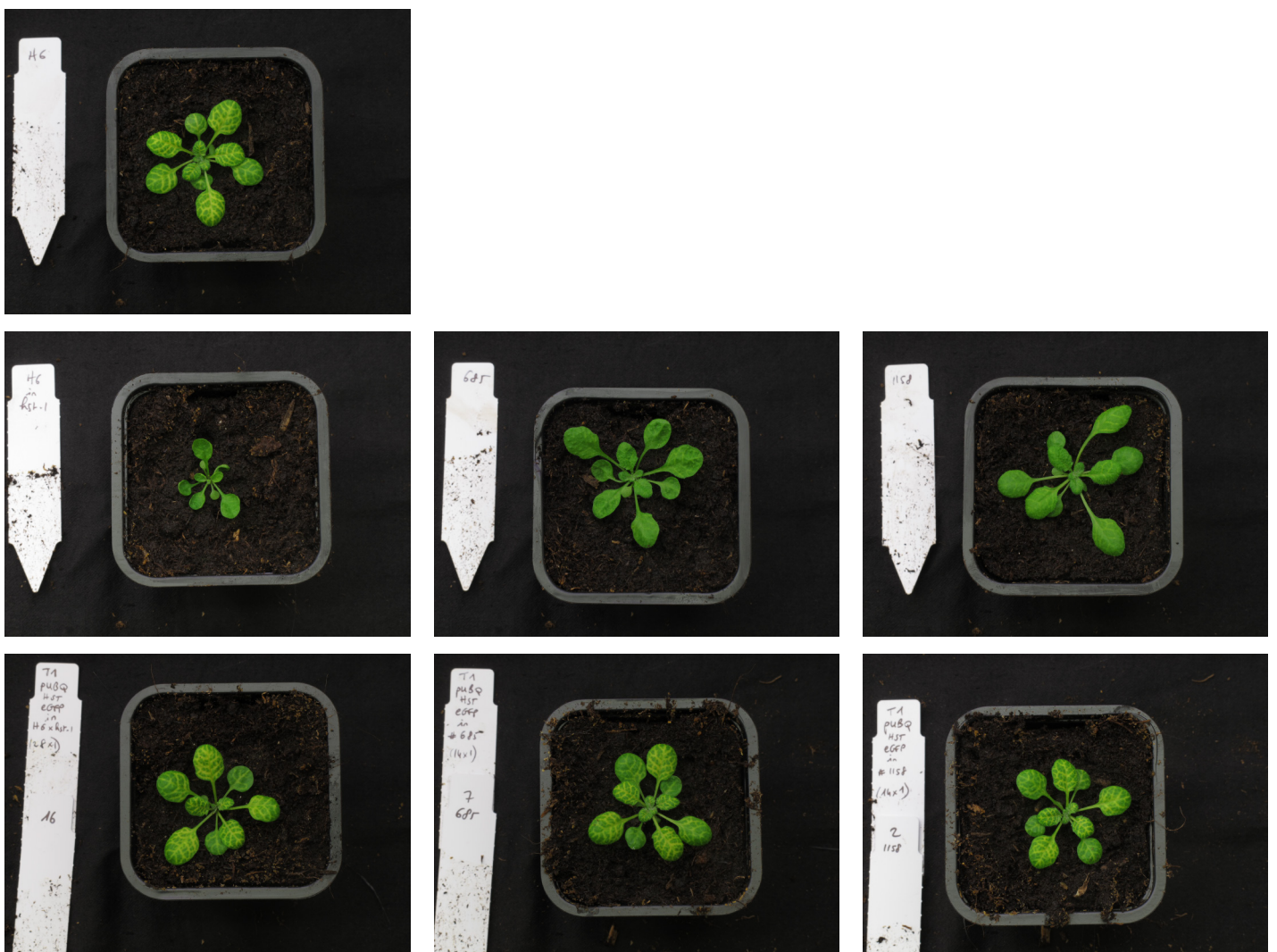

Supplement: Supplementary file 3 — Source Data for Appendix [file EMBJ-40-e107455-s006.zip › Appendix Figure S6 Source Data.pdf]

Appendix Figure S7

HST

Left panel

Right panel

Coom Left

Coom Right

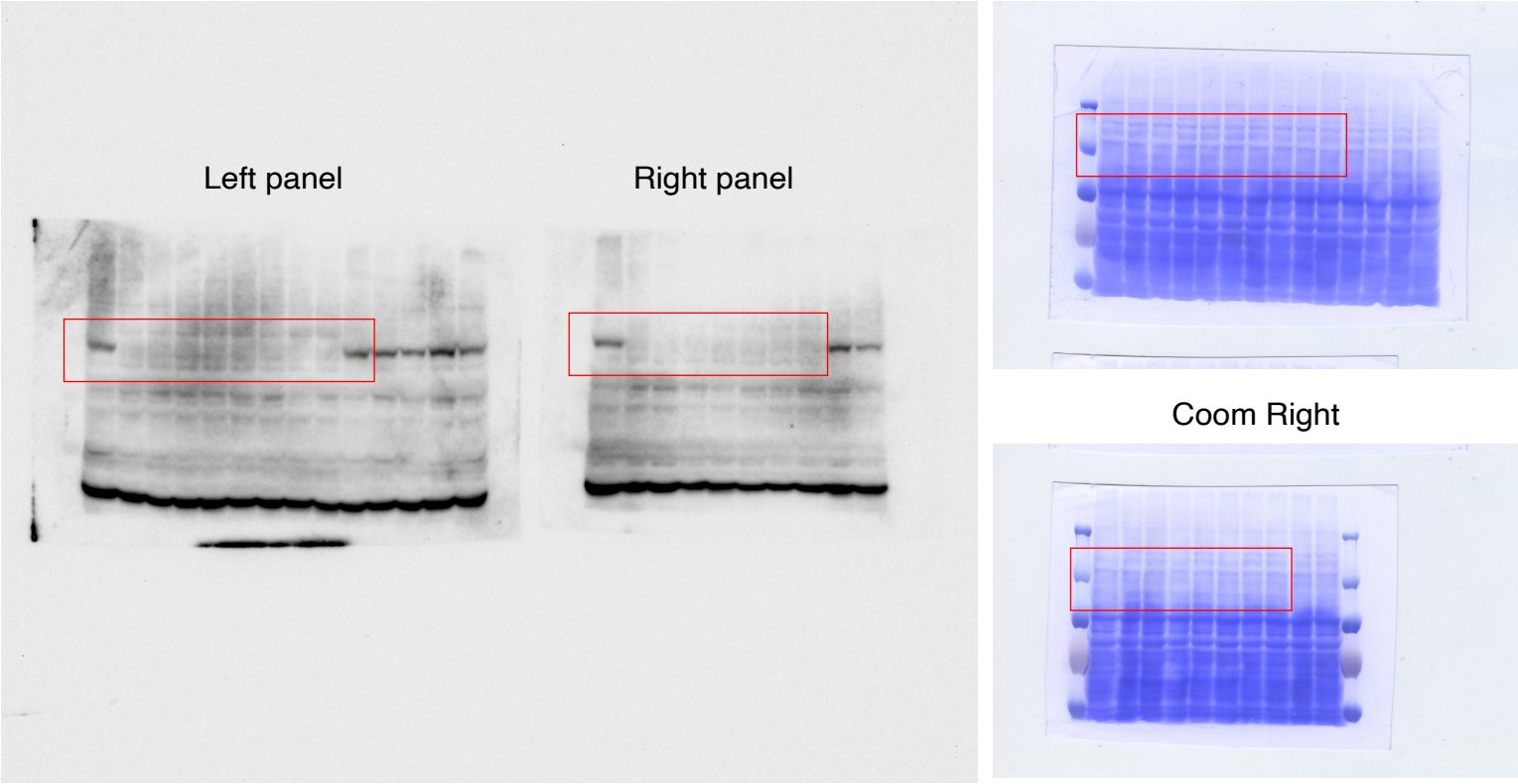

Supplement: Supplementary file 3 — Source Data for Appendix [file EMBJ-40-e107455-s006.zip › Appendix Figure S7 Source Data.pdf]

Appendix Figure S2

BF

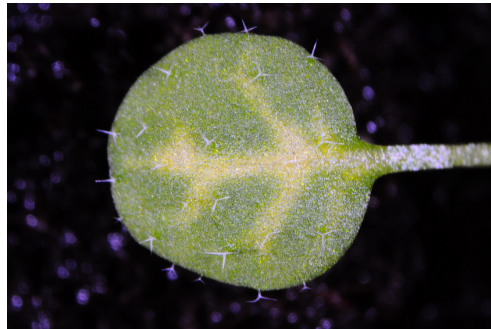

GFP

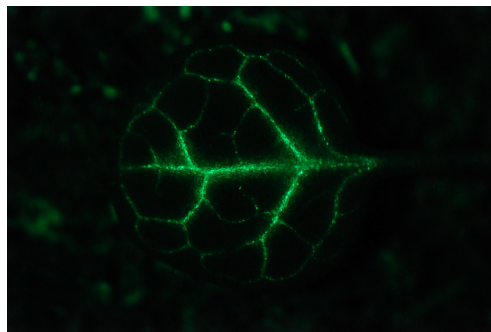

subtraction

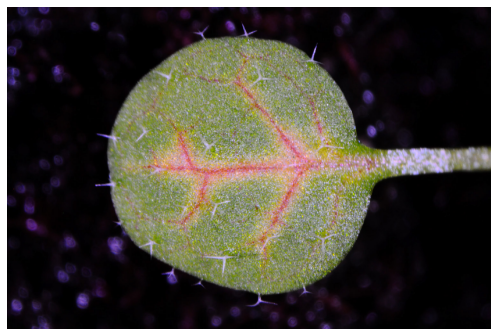

Supplement: Supplementary file 3 — Source Data for Appendix [file EMBJ-40-e107455-s006.zip › Appendix Figure S2 Source Data.pdf]

Appendix Figure S5

HST

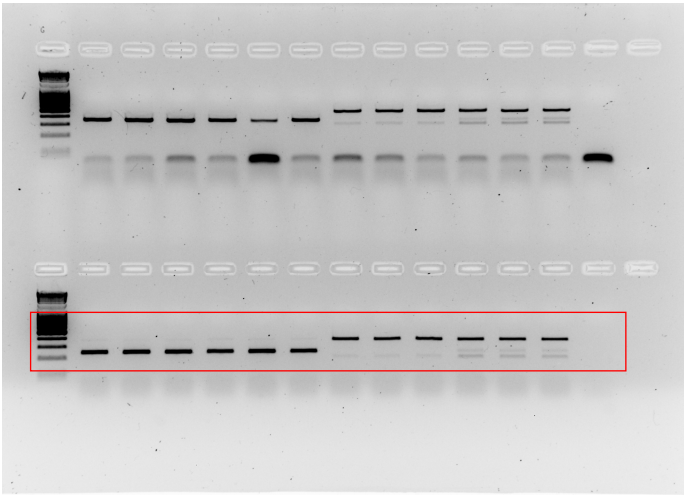

TCTP

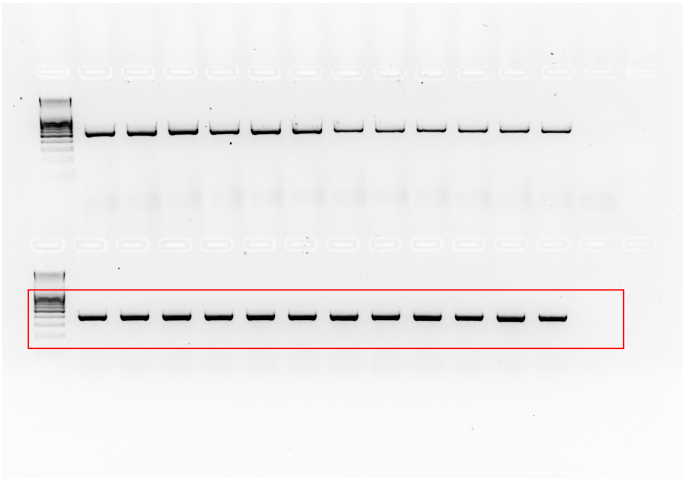

Supplement: Supplementary file 3 — Source Data for Appendix [file EMBJ-40-e107455-s006.zip › Appendix Figure S5 Source Data.pdf]

Appendix Figure S14

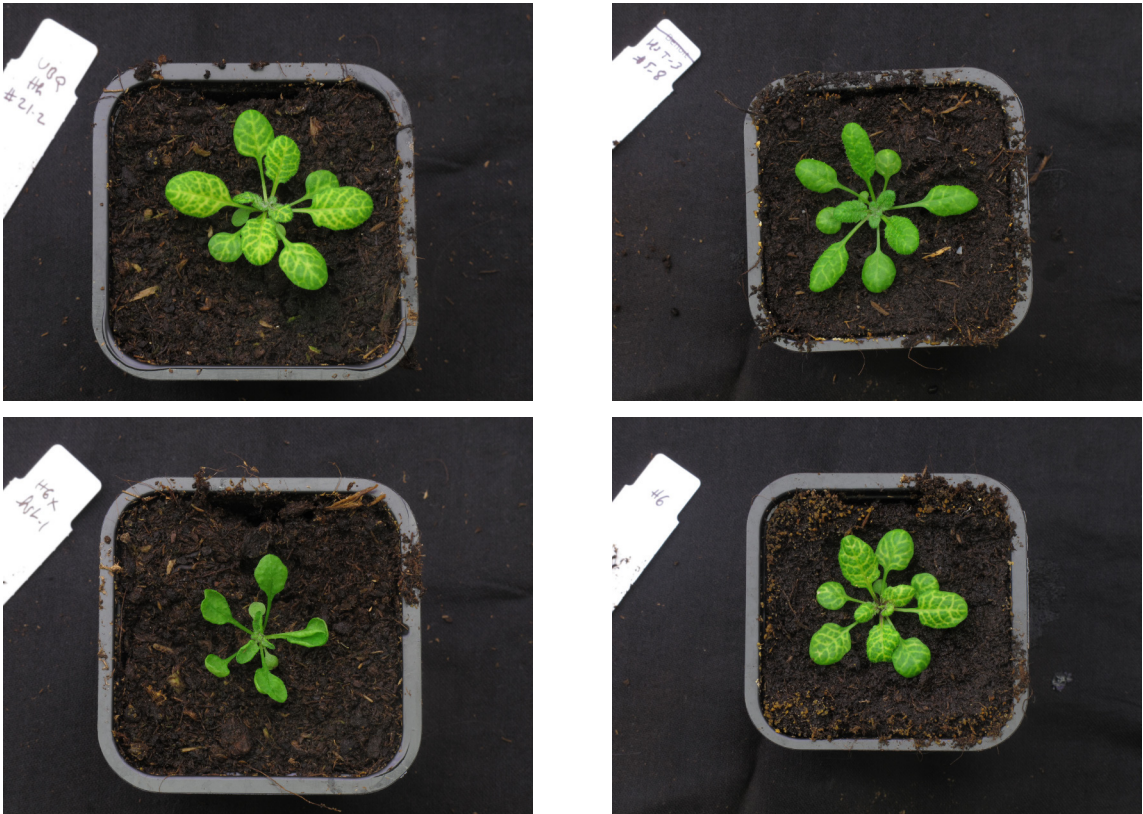

HST

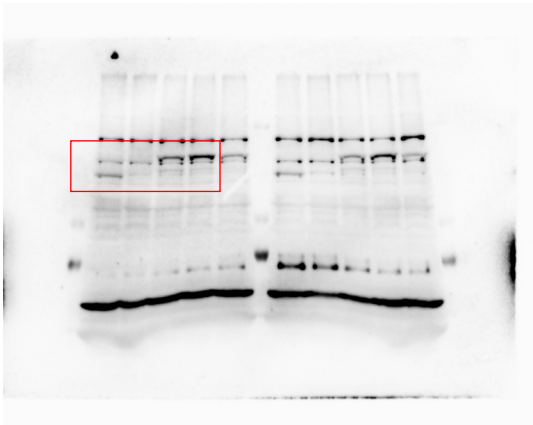

Coom

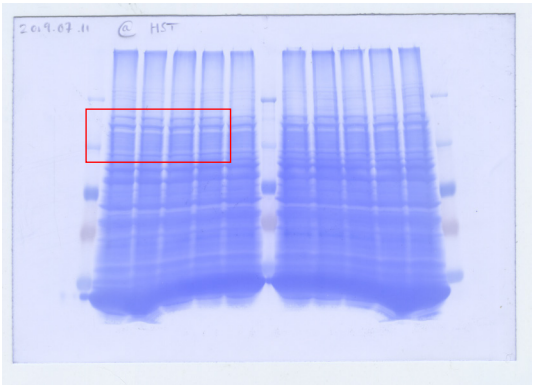

Supplement: Supplementary file 3 — Source Data for Appendix [file EMBJ-40-e107455-s006.zip › Appendix Figure S14 Source Data.pdf]

Appendix Figure S4B

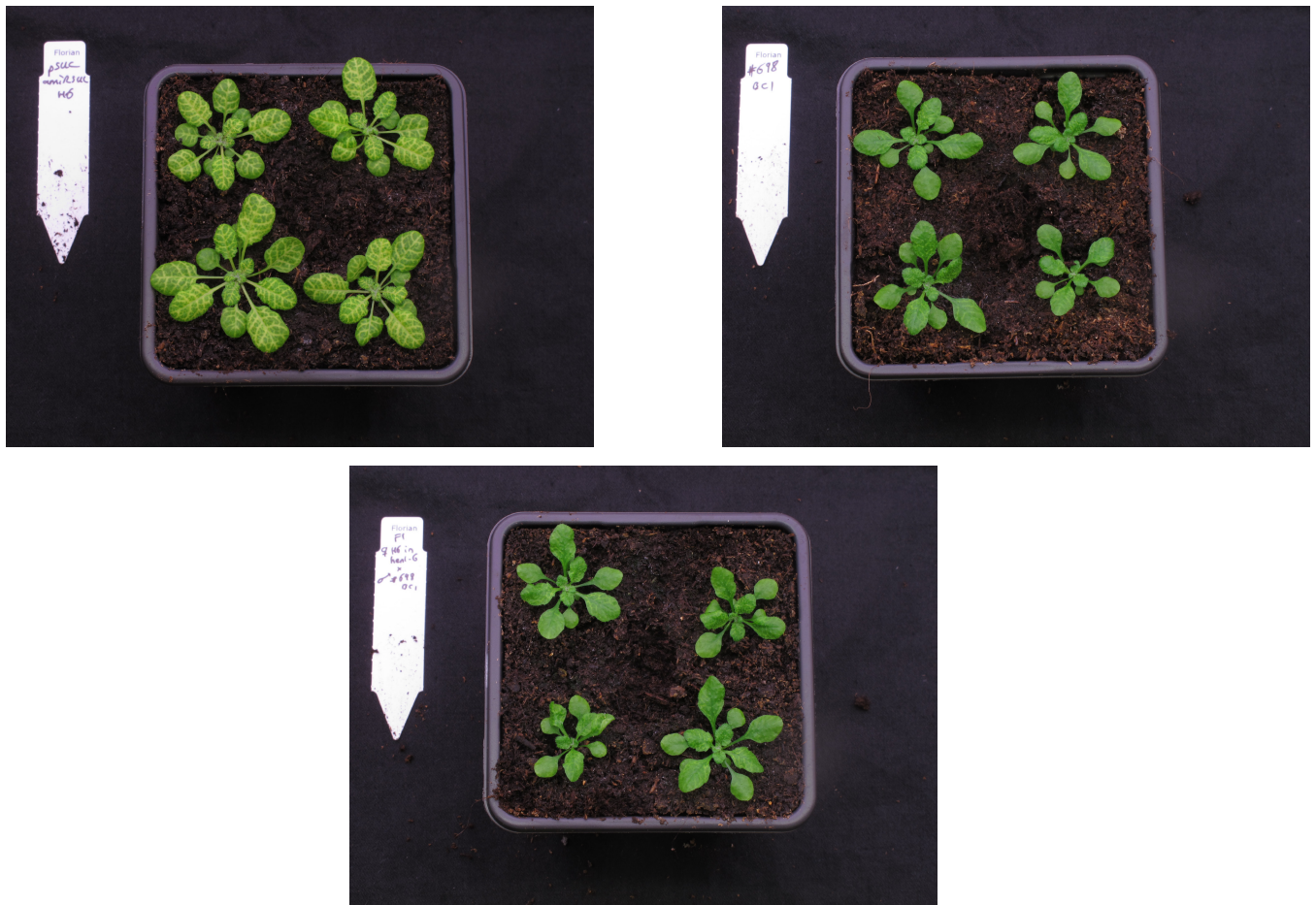

Appendix Figure S4C

amiRSUL

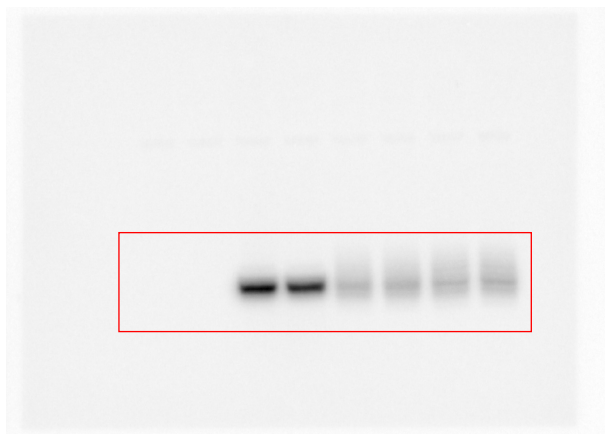

miR171

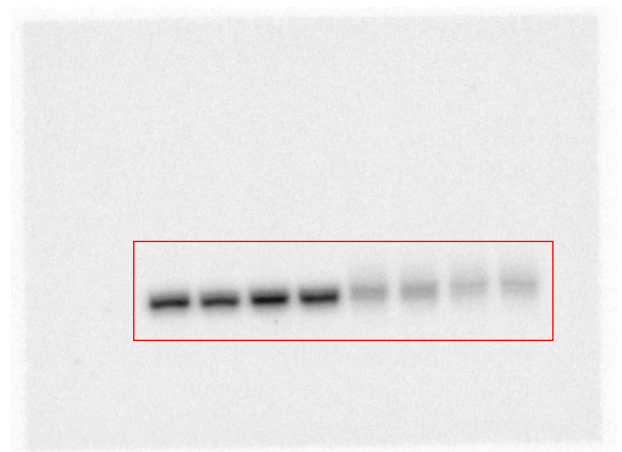

U6

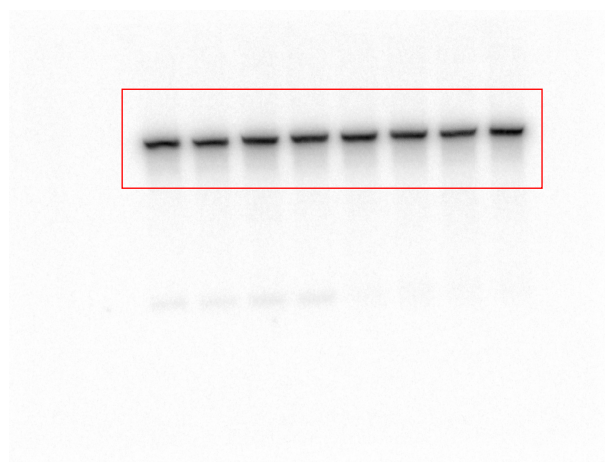

Supplement: Supplementary file 3 — Source Data for Appendix [file EMBJ-40-e107455-s006.zip › Appendix Figure S4 Source Data.pdf]

Appendix Figure S13A

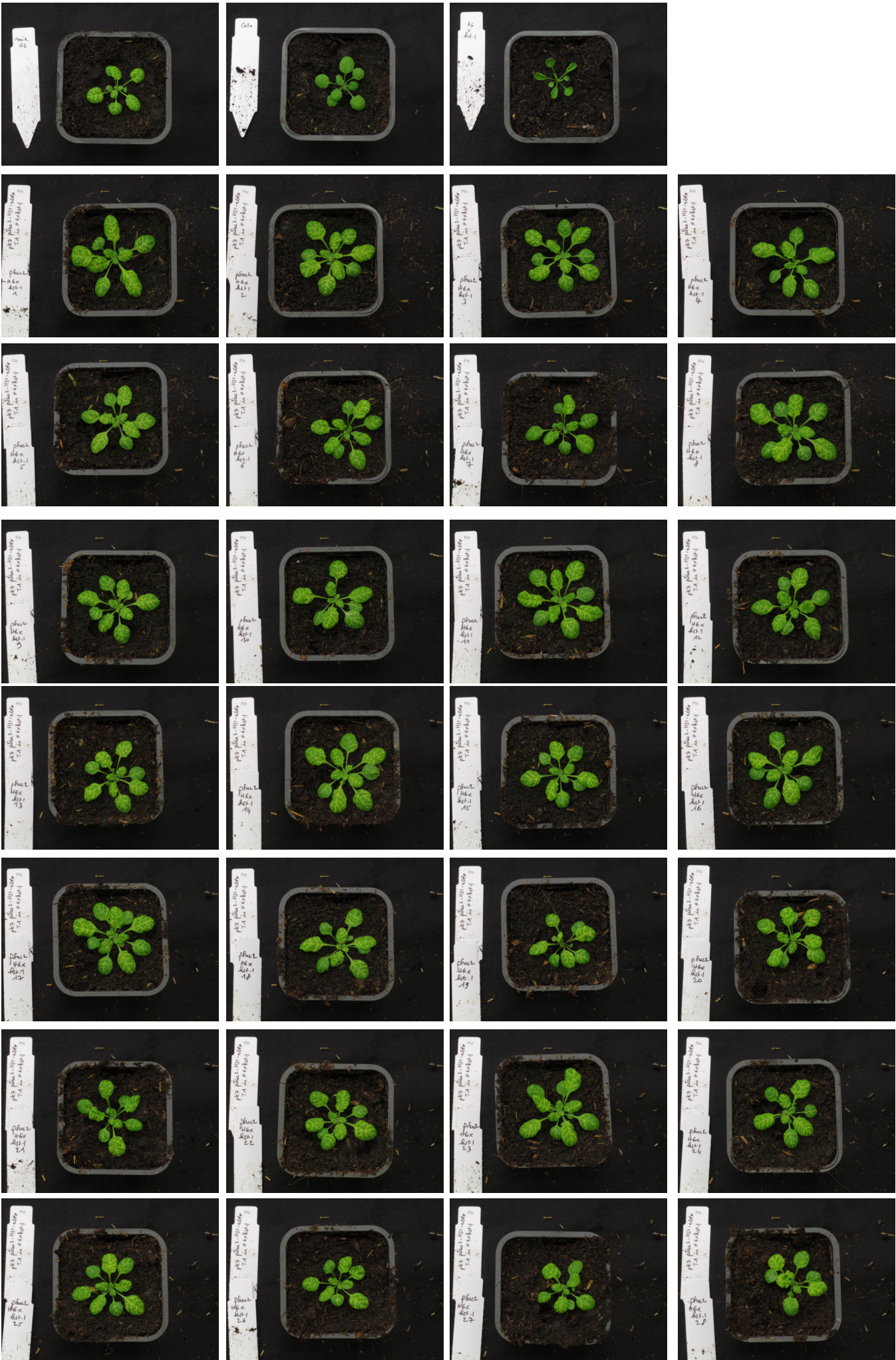

Appendix Figure S13B

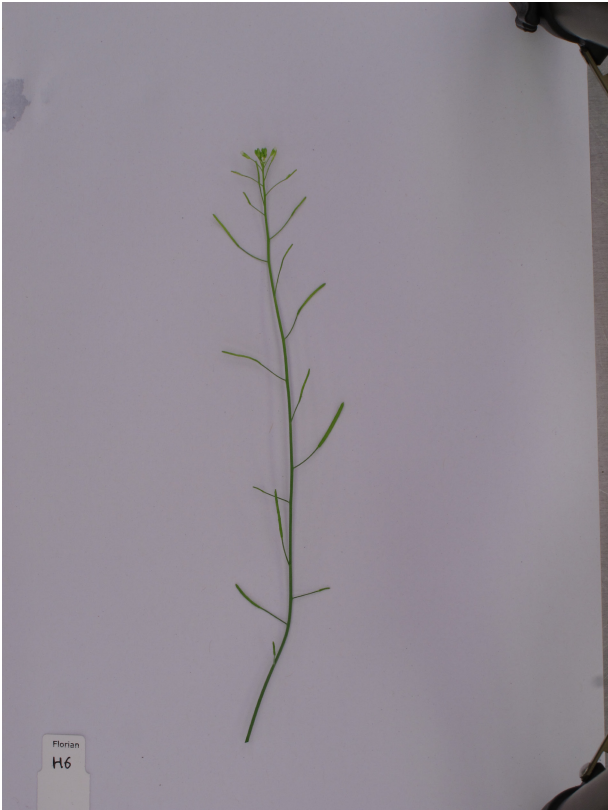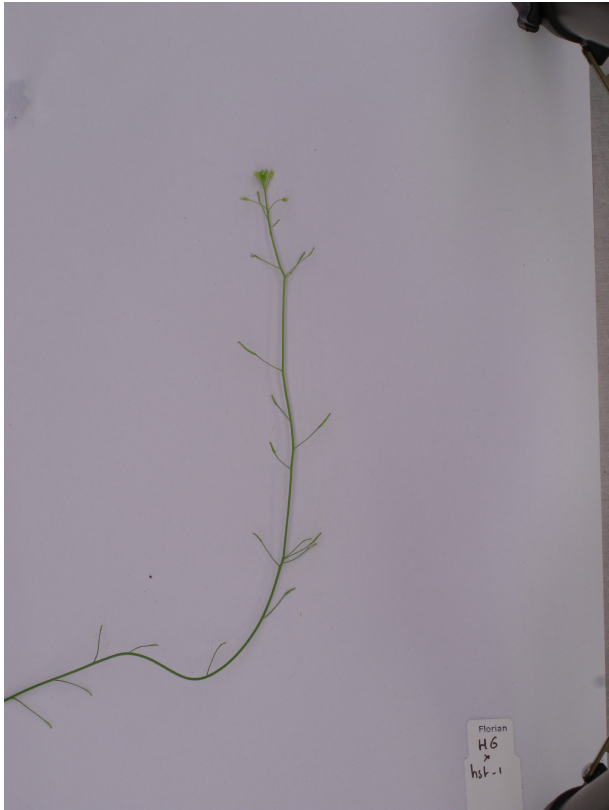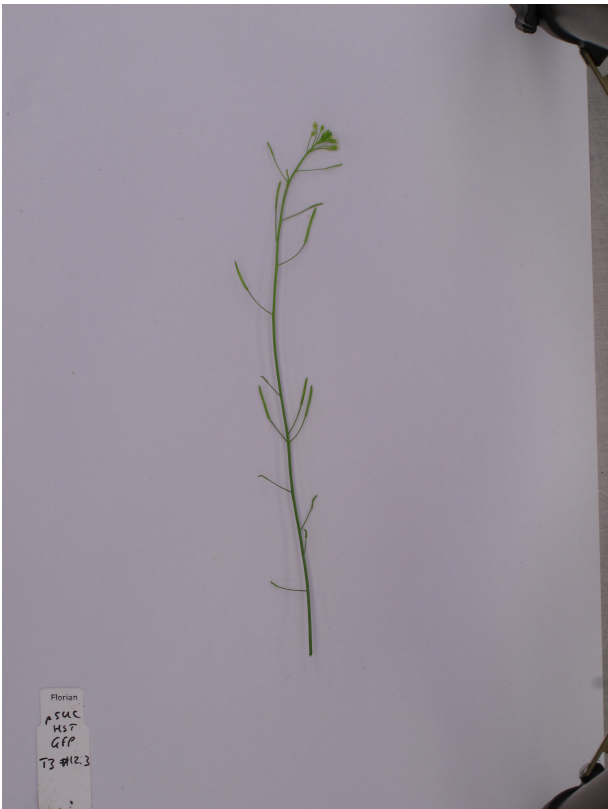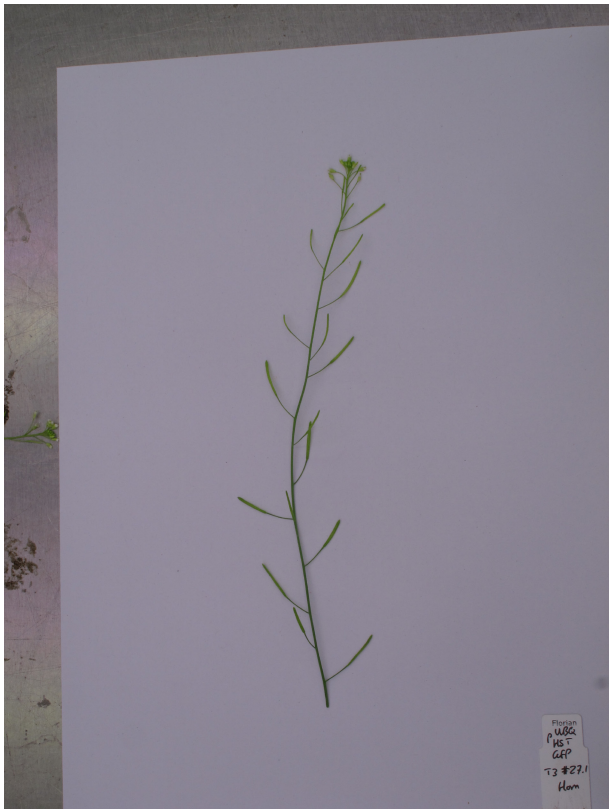

Supplement: Supplementary file 3 — Source Data for Appendix [file EMBJ-40-e107455-s006.zip › Appendix Figure S13 Source Data.pdf]

Figure 1B

WT

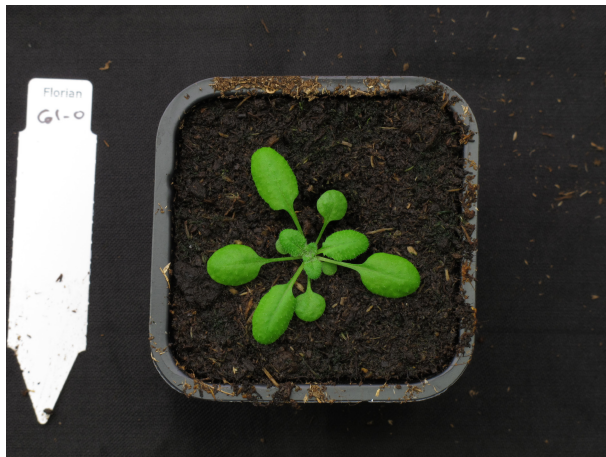

pSUC2::amiRSUL

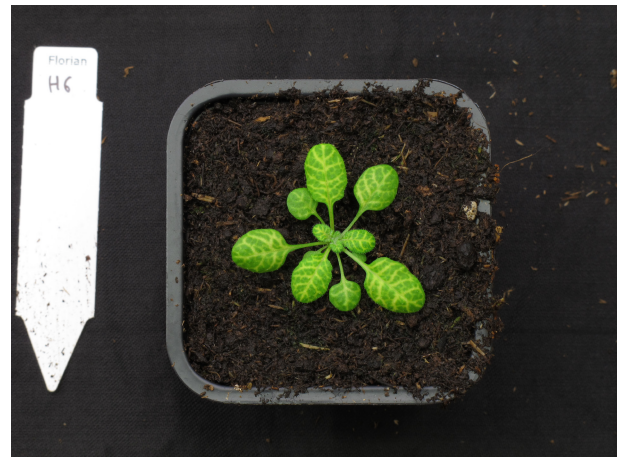

Figure 1C

WT

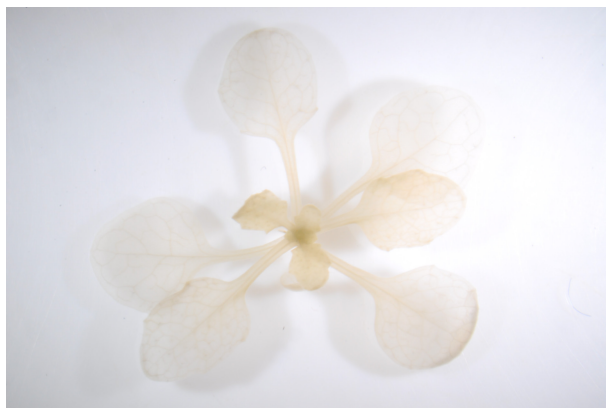

pSUC2::GUS

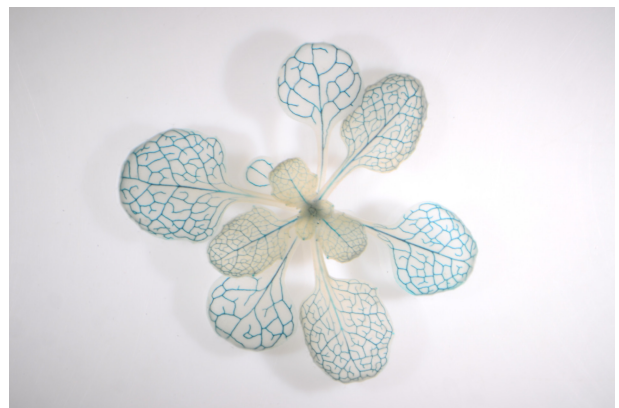

Figure 1E

amiRSUL

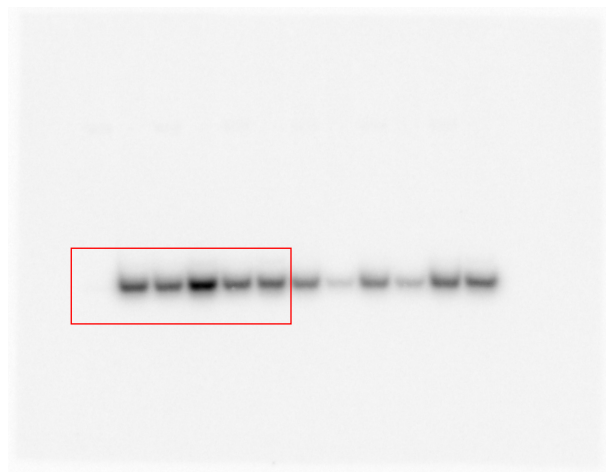

U6

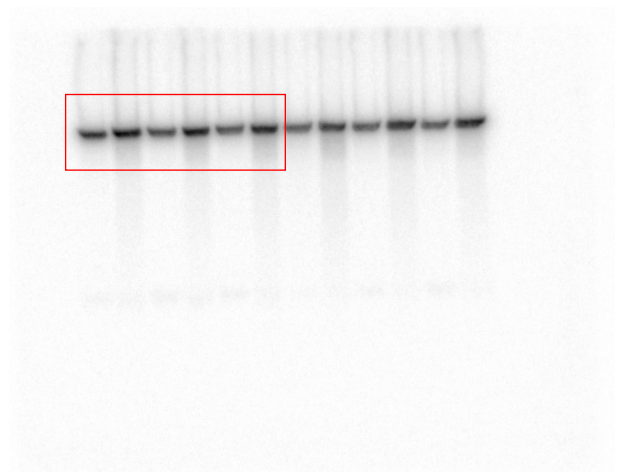

Figure 1F

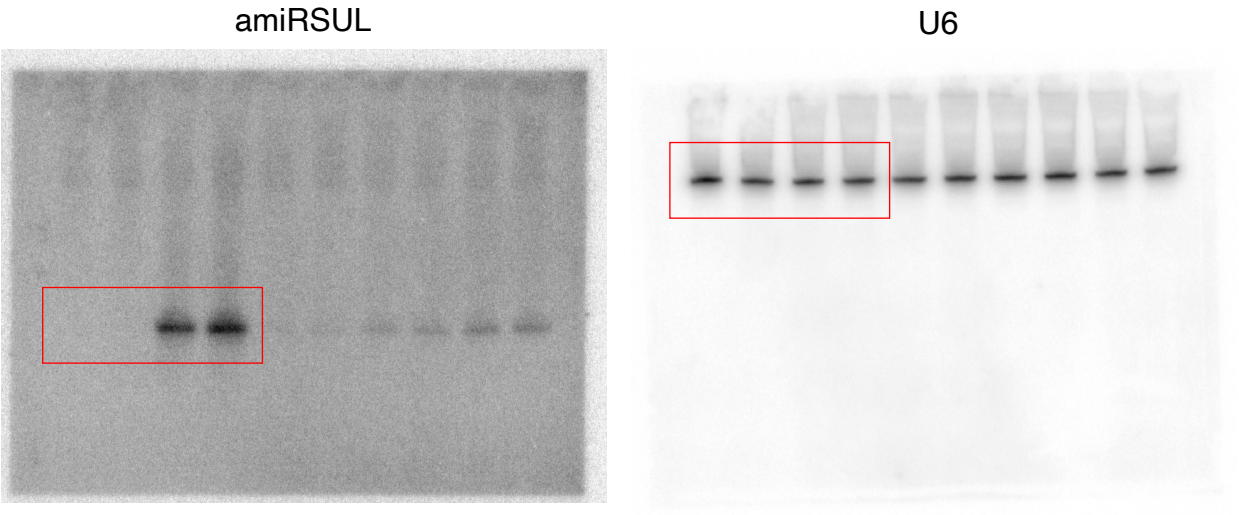

Figure 1G

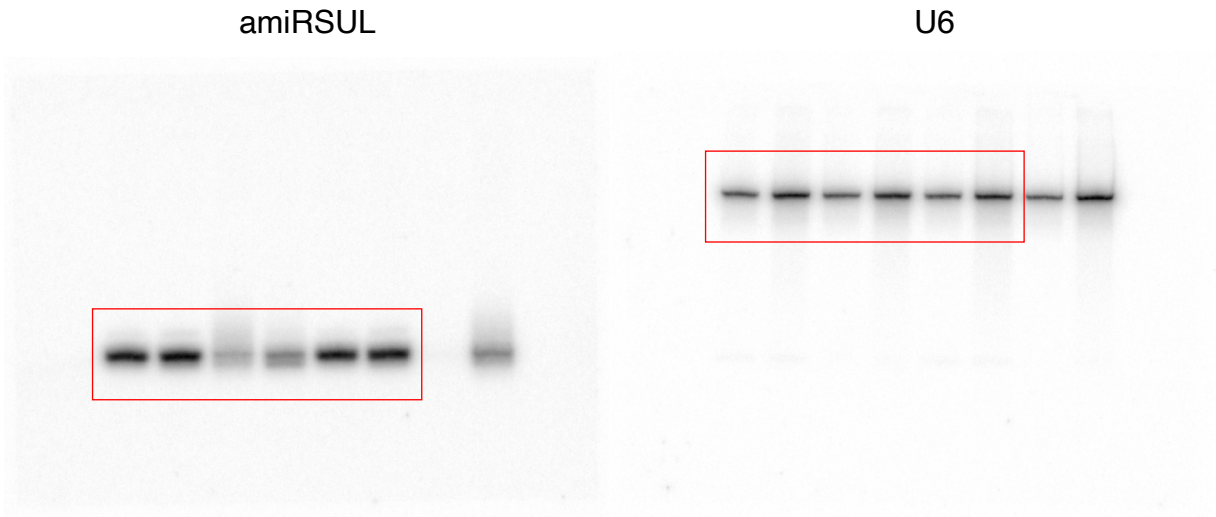

Supplement: Supplementary file 4 — Source Data for Figure 1 [file EMBJ-40-e107455-s005.zip › Raw_data_Fig_1.pdf]

Figure 2B

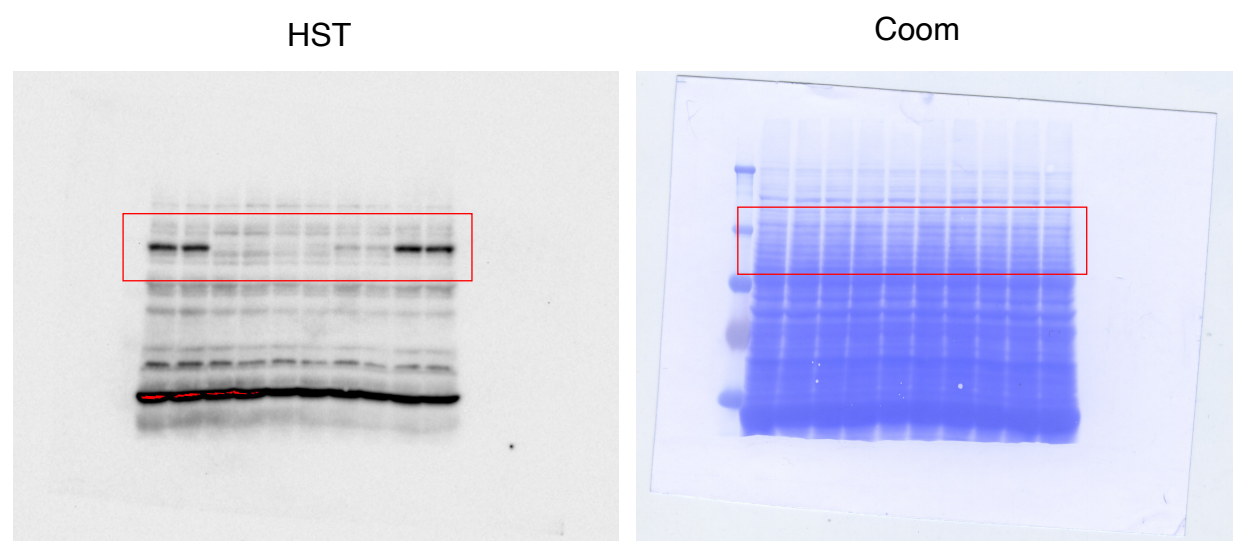

Figure 2C

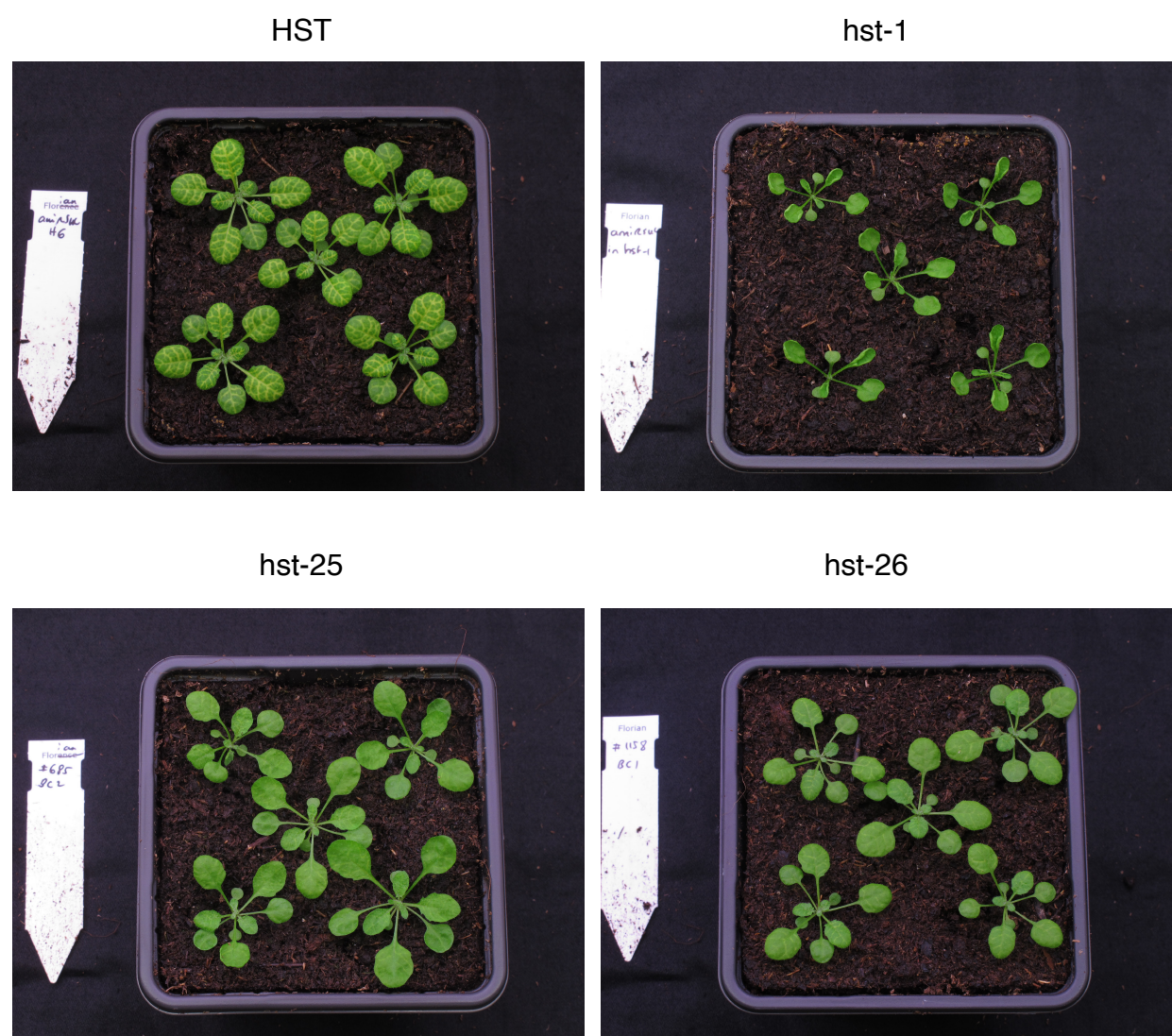

Supplement: Supplementary file 5 — Source Data for Figure 2 [file EMBJ-40-e107455-s004.zip › Raw_data_Fig_2.pdf]

Figure 4A

Rep#1

Rep#2

amiRSUL

amiRSUL

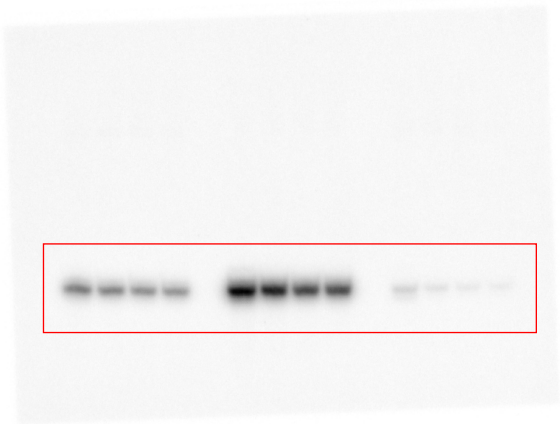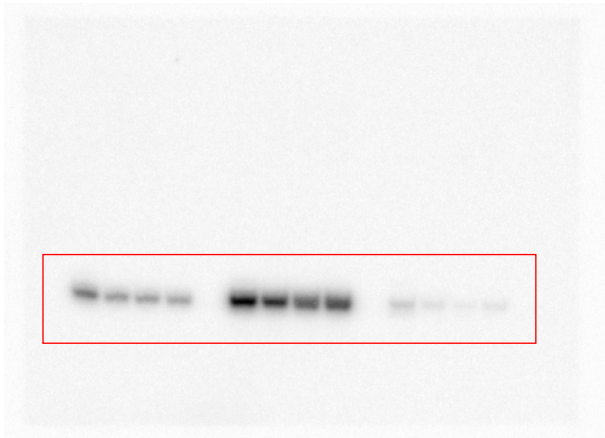

miR165

miR165

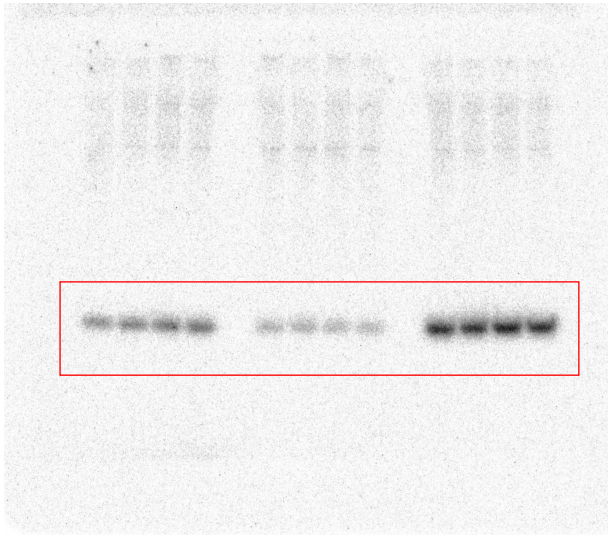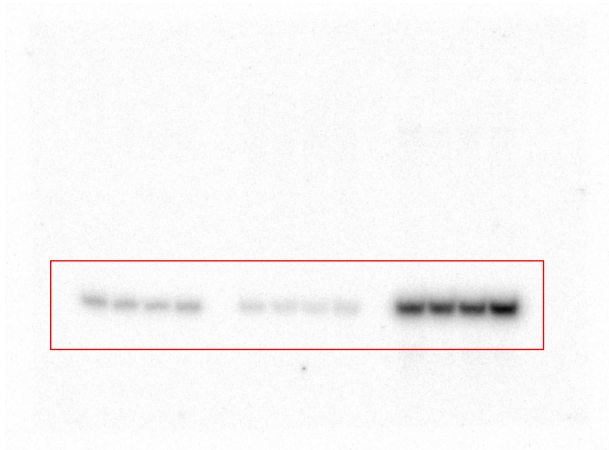

U6

U6

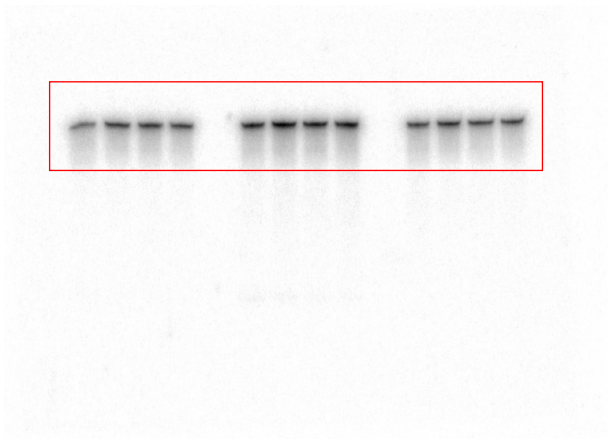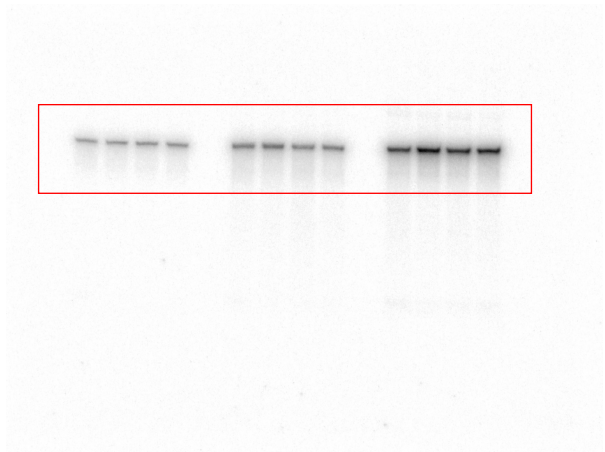

Figure 4C

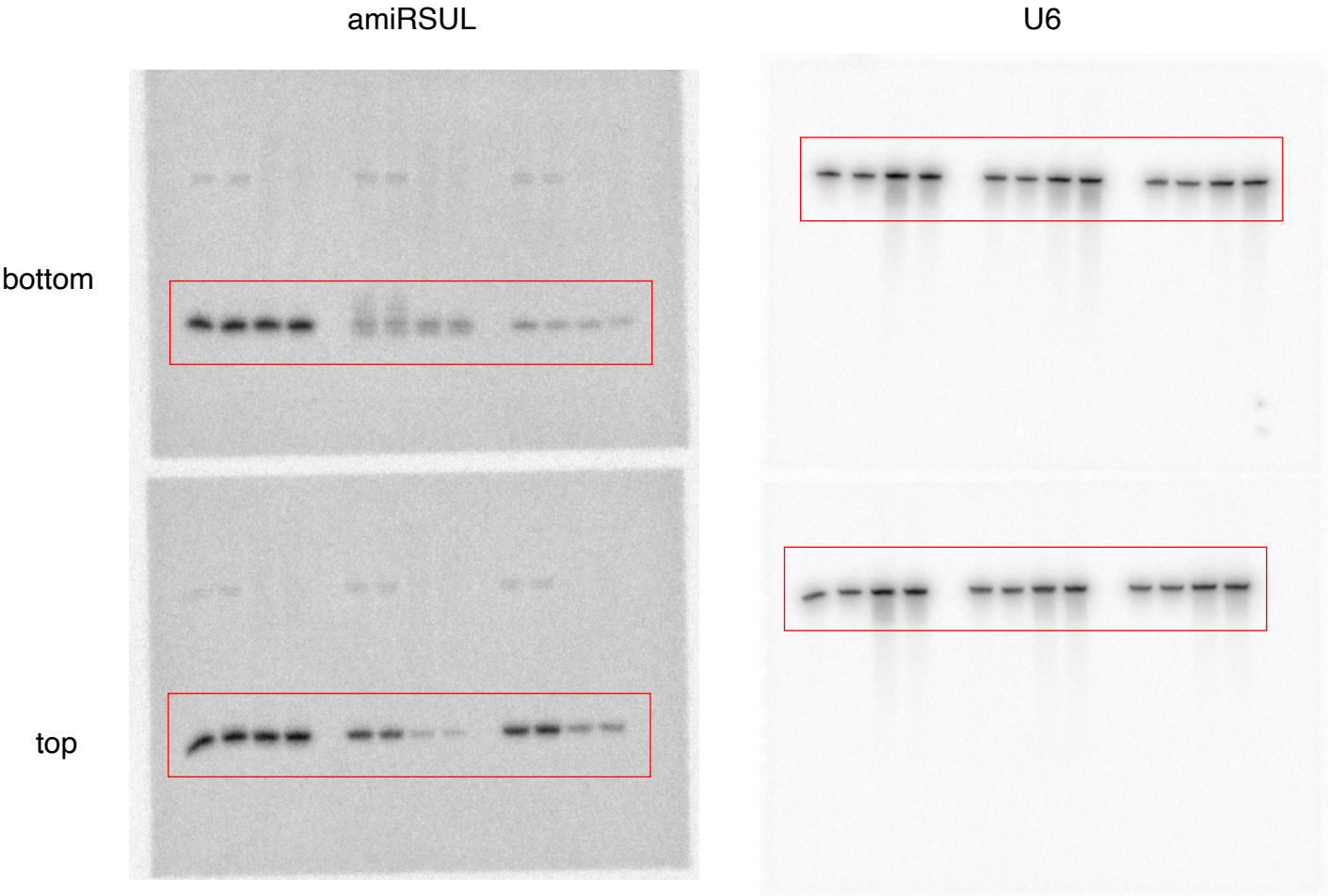

Figure 4E

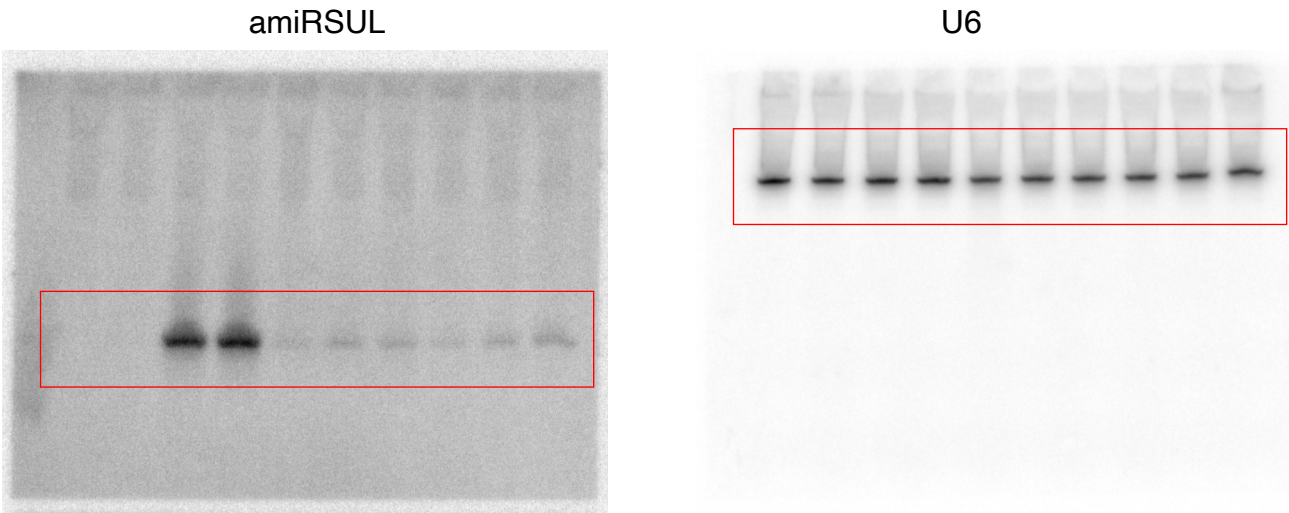

Supplement: Supplementary file 7 — Source Data for Figure 4 [file EMBJ-40-e107455-s002.zip › Raw_data_Fig_4.pdf]

Figure 5A

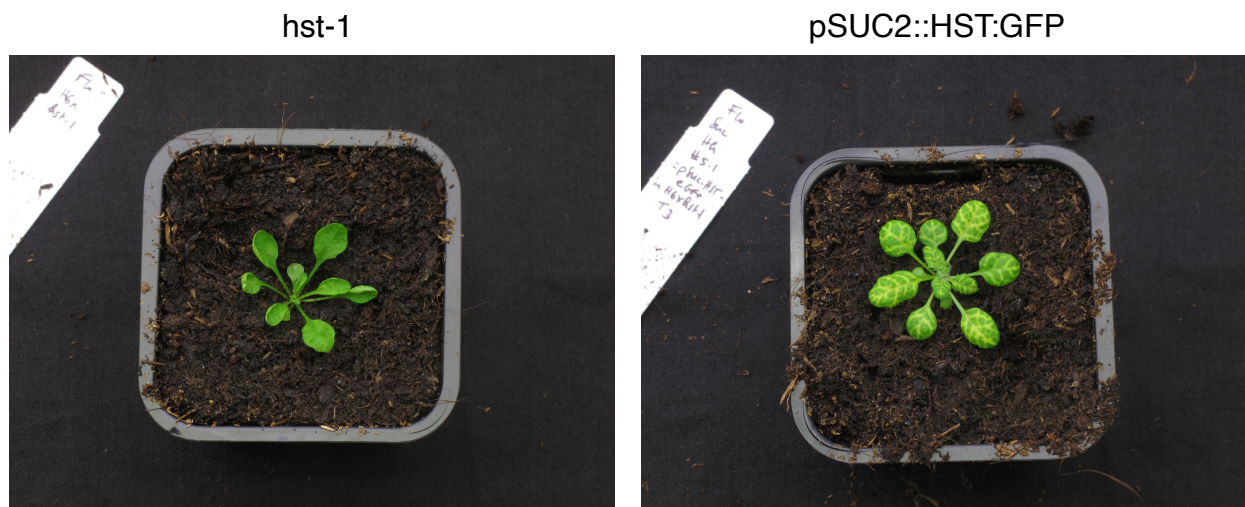

Figure 5B

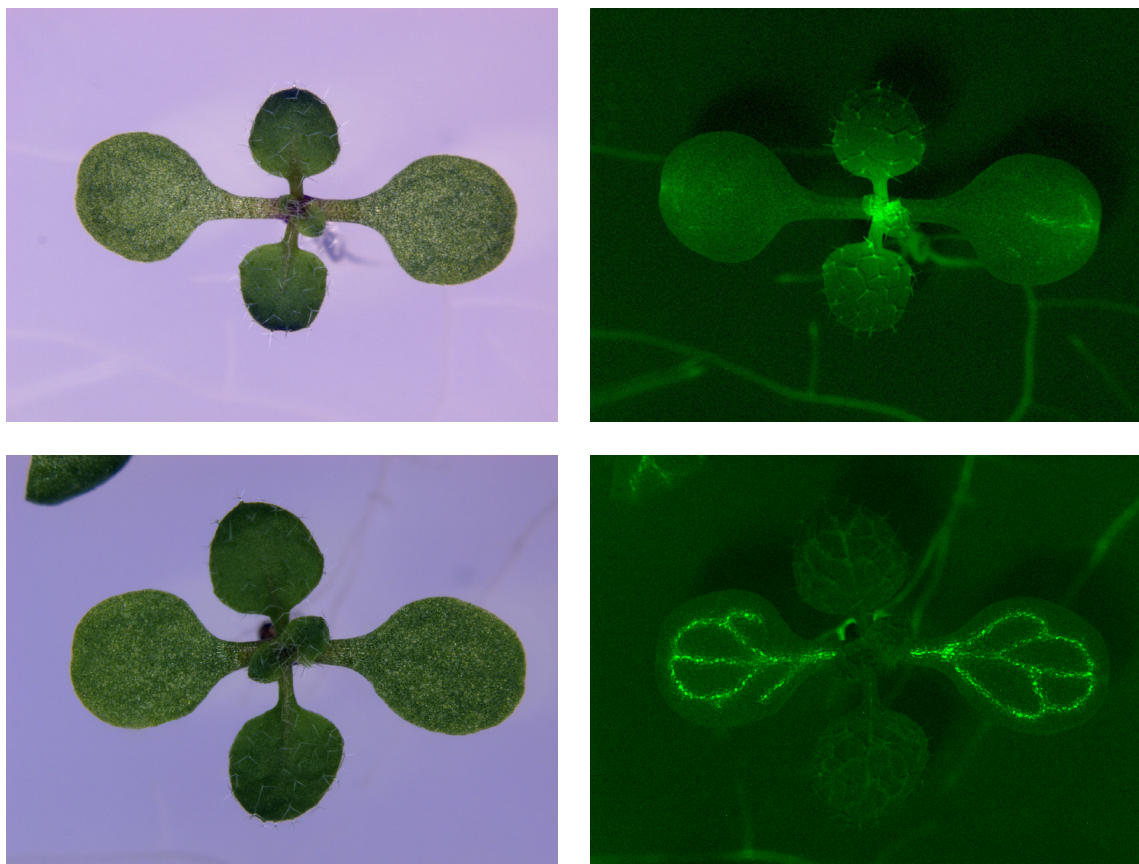

Figure 5C

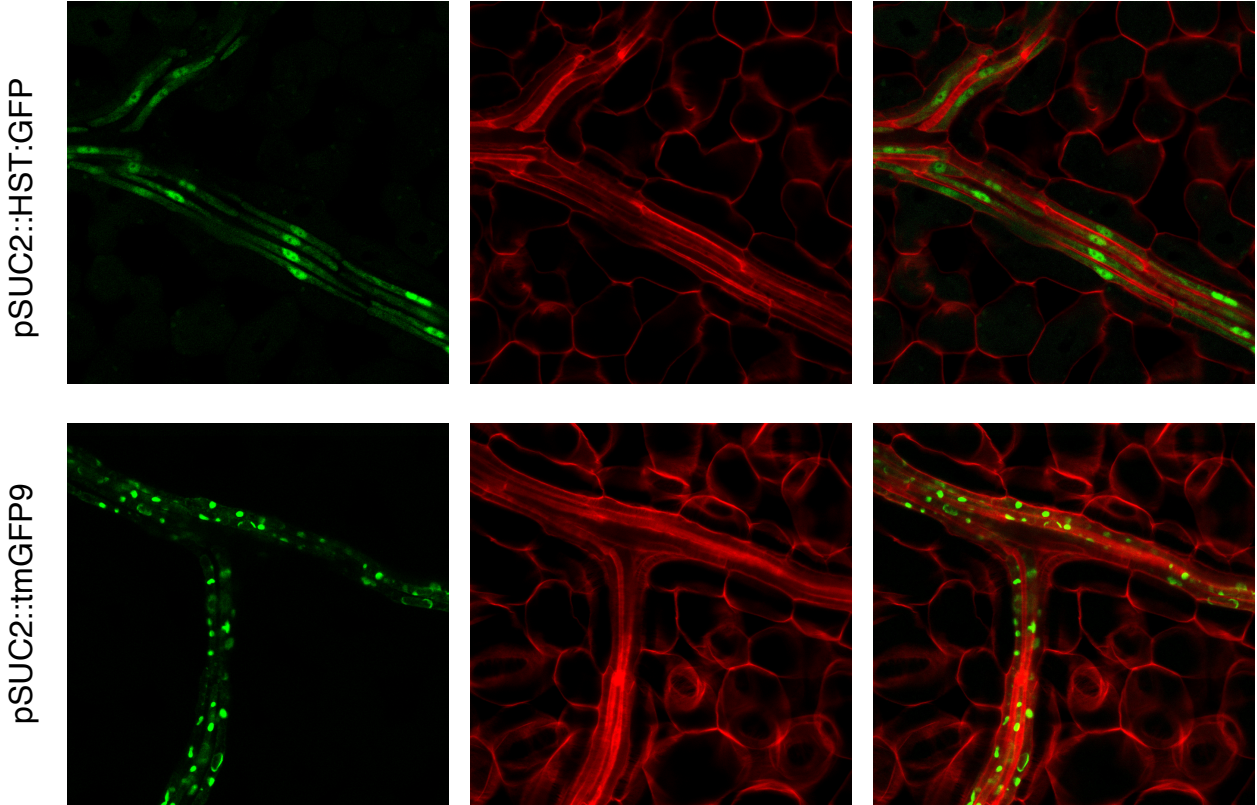

Figure 5D

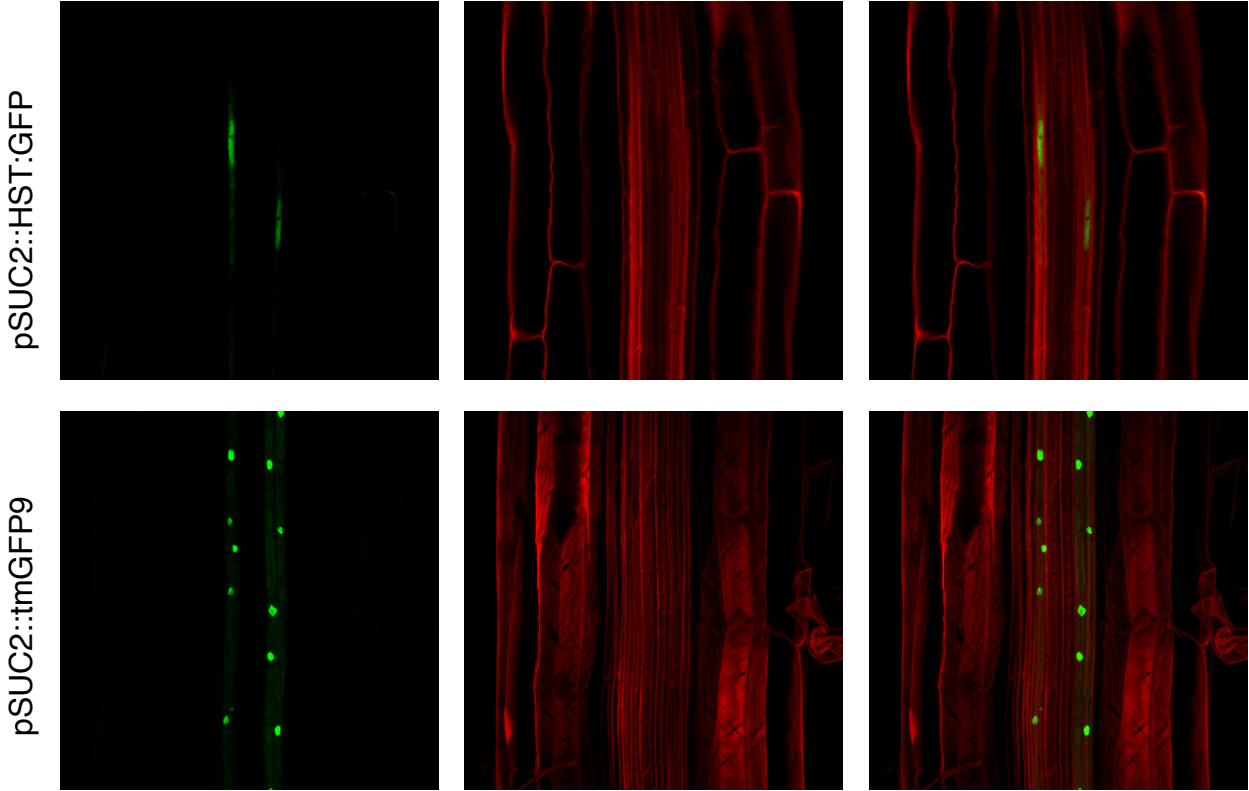

Figure 5E

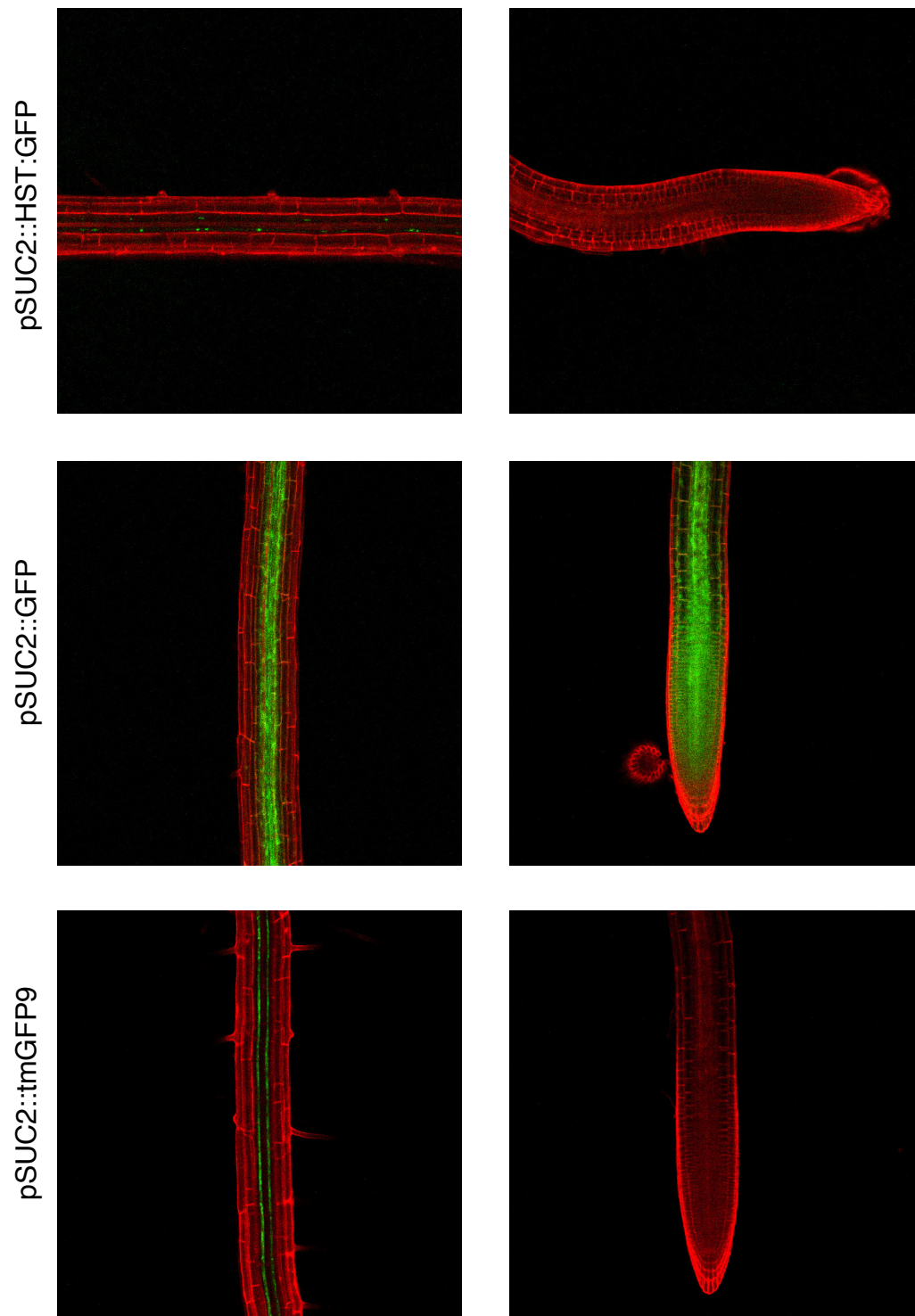

Figure 5F

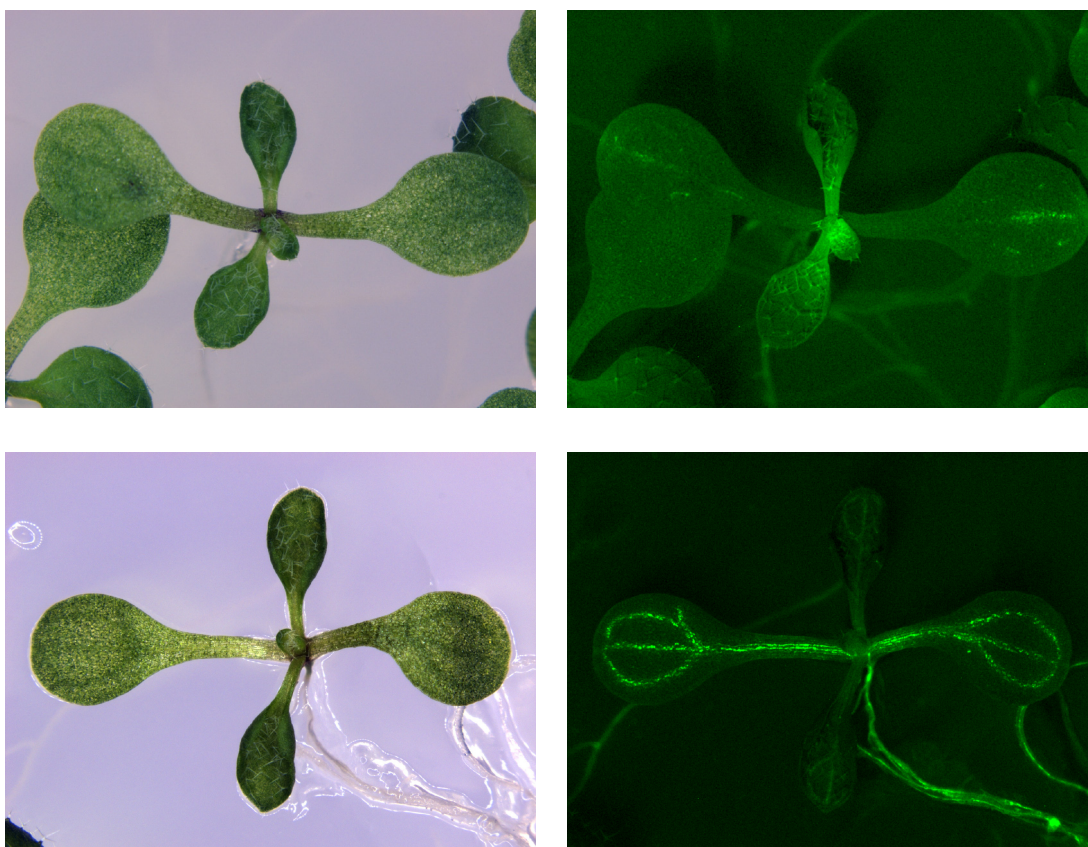

Figure 5G

pSUC2::GFP x *hst-1*

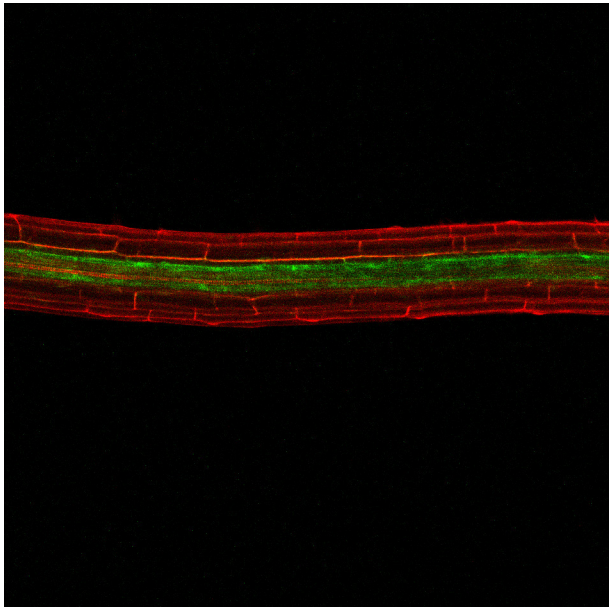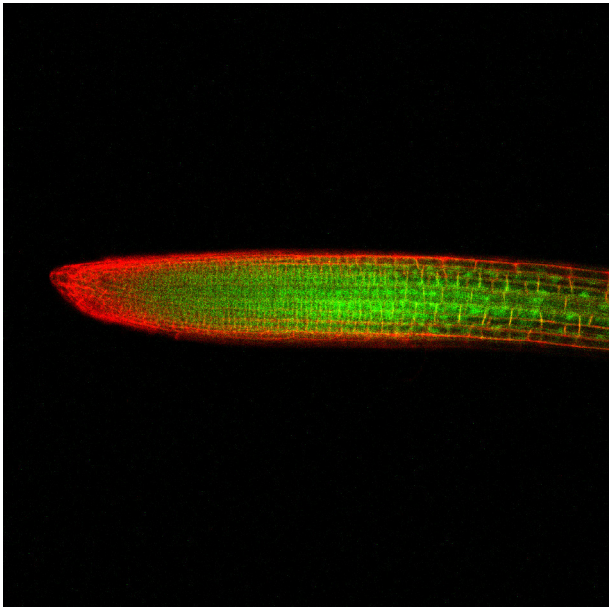

pSUC2::tmGFP9 x *hst-1*

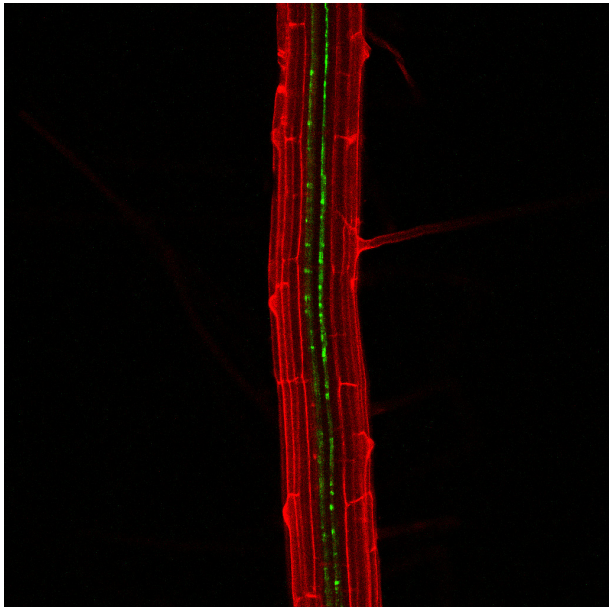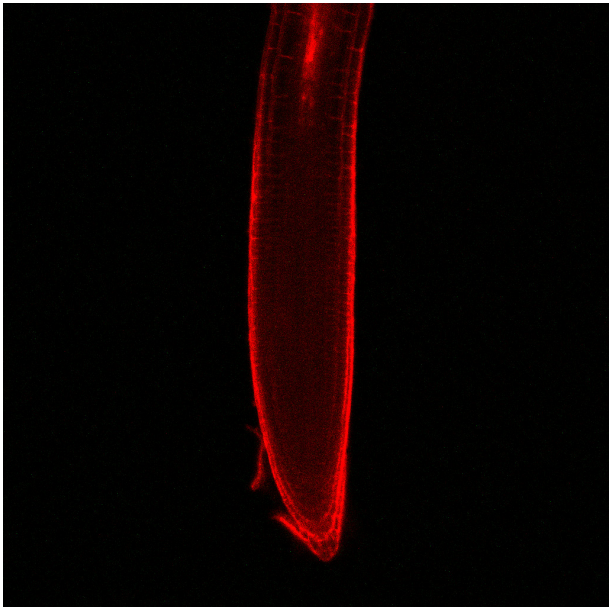

Supplement: Supplementary file 8 — Source Data for Figure 5 [file EMBJ-40-e107455-s001.pdf]

Figure 6A

(i)

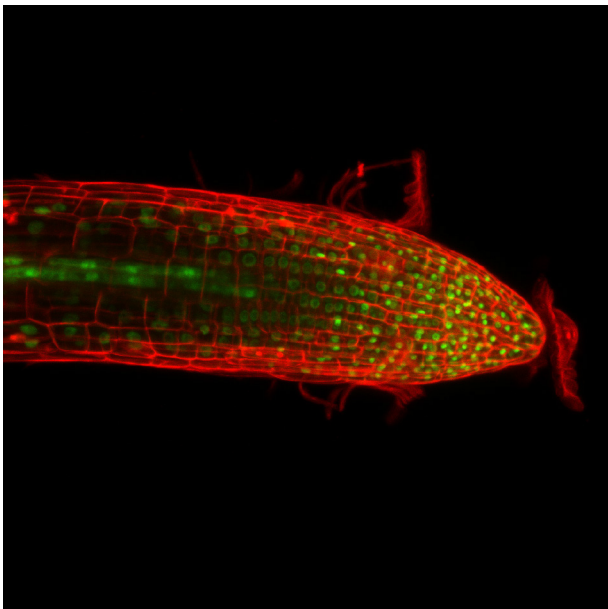

(ii)

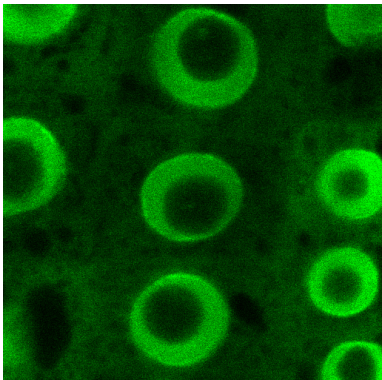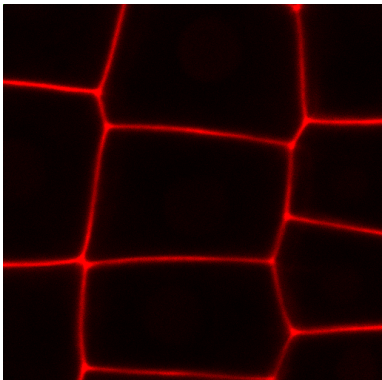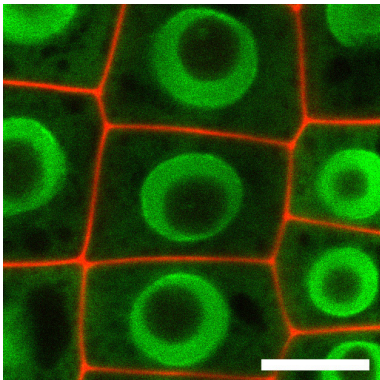

(iii)

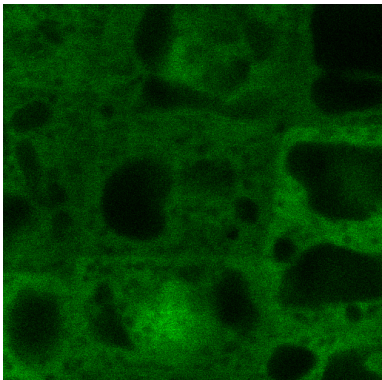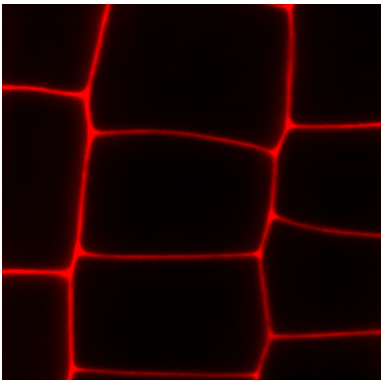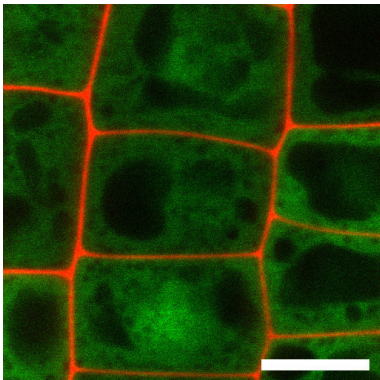

Figure 6B

HST

hst-3

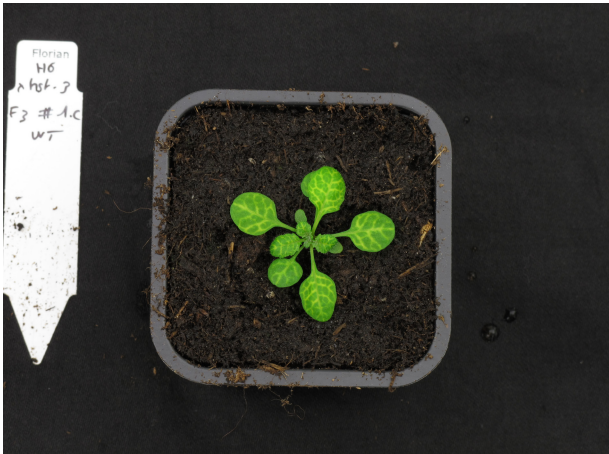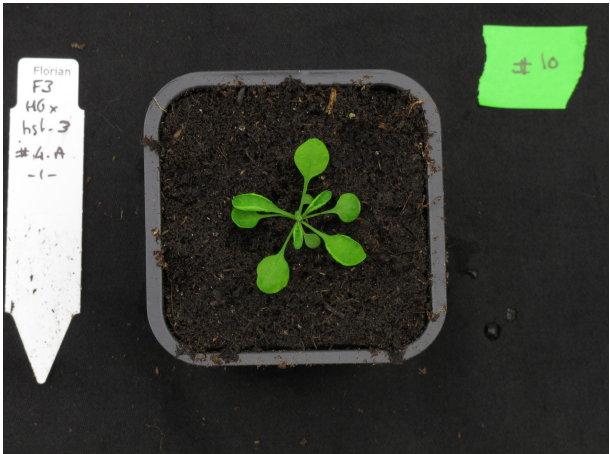

Figure 6C

(i)

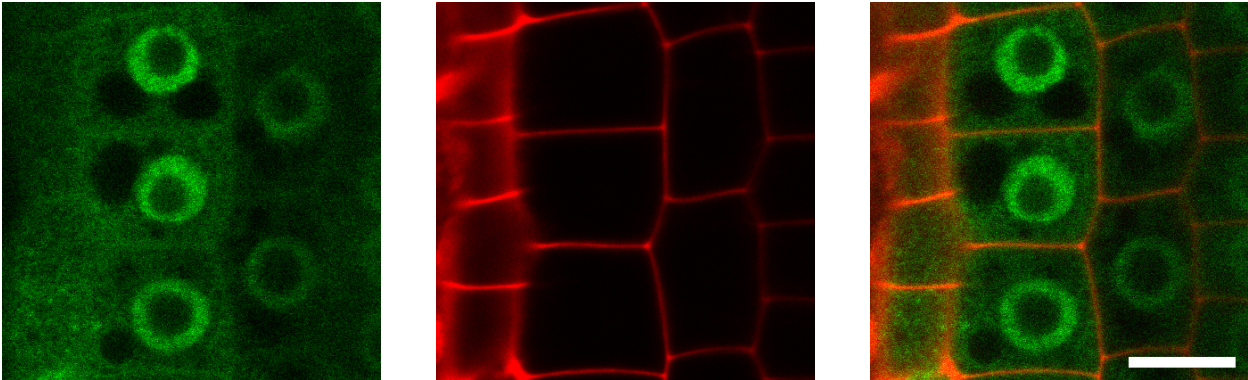

(ii)

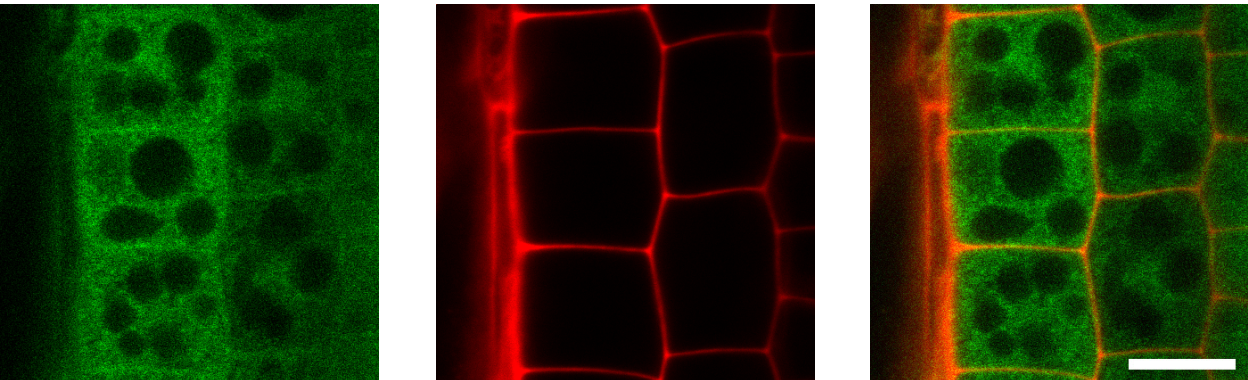

Figure 6D

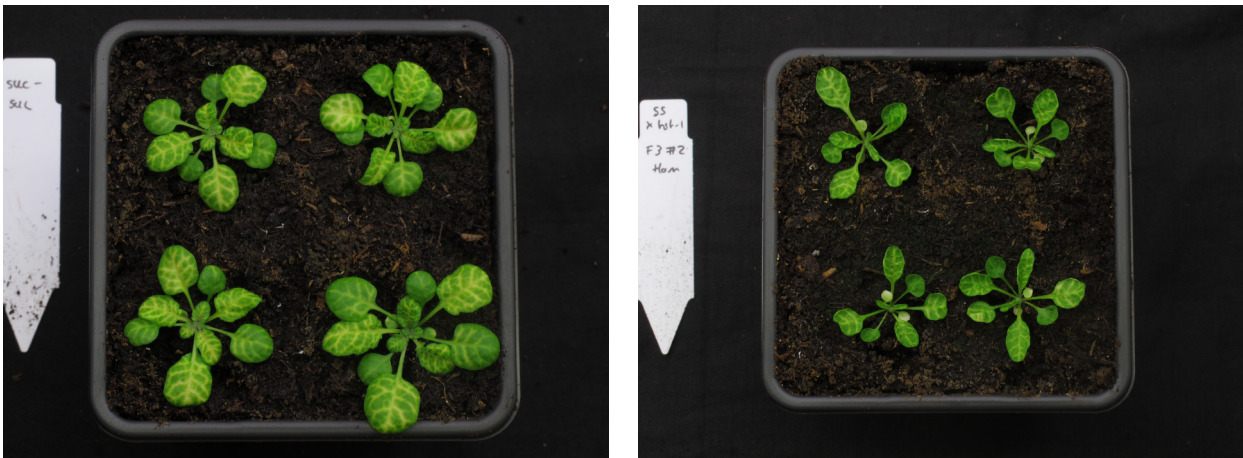

Supplement: Supplementary file 9 — Source Data for Figure 6 [file EMBJ-40-e107455-s007.pdf]

Figure 7A

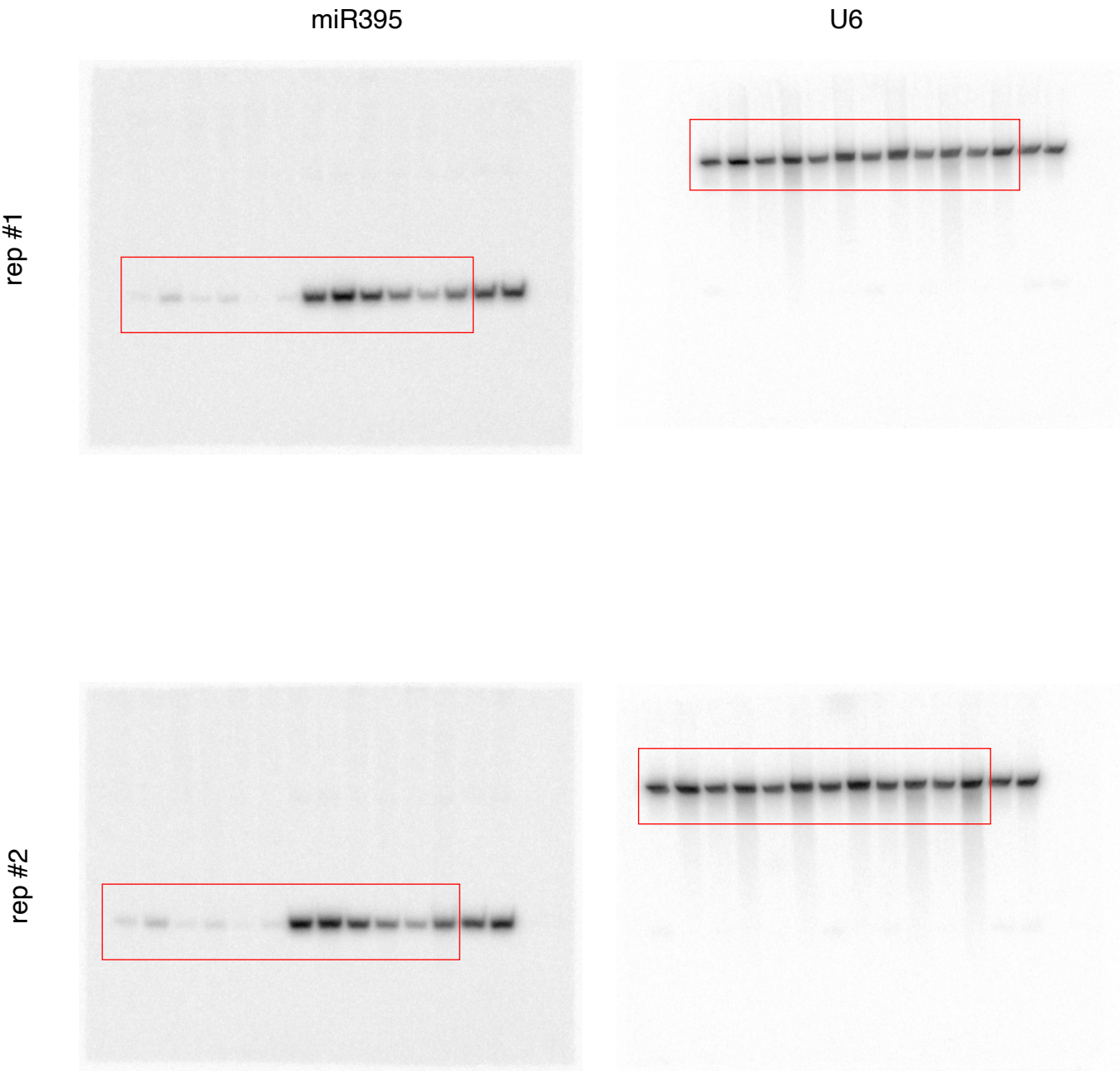

Figure 7C

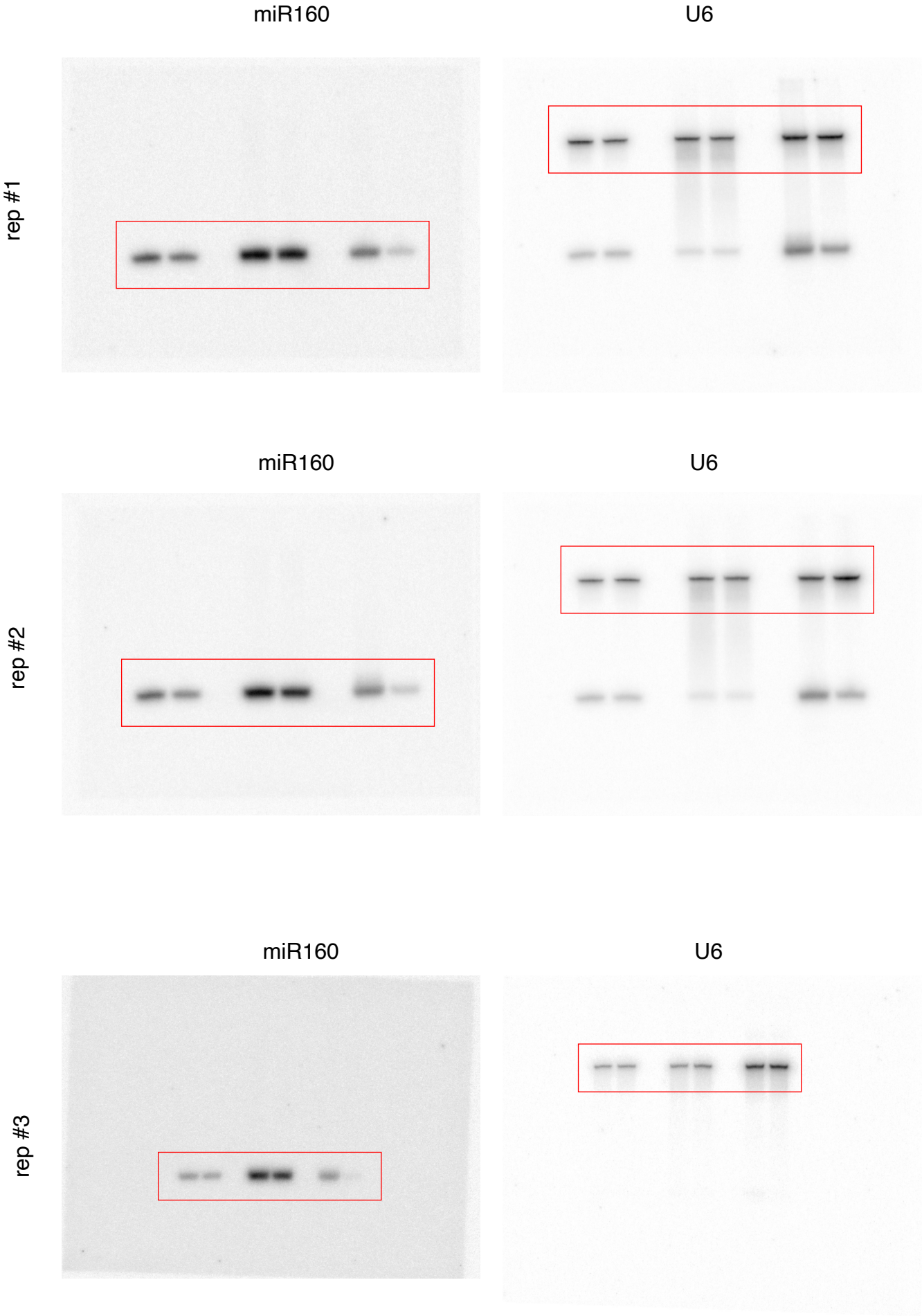

Figure 7E

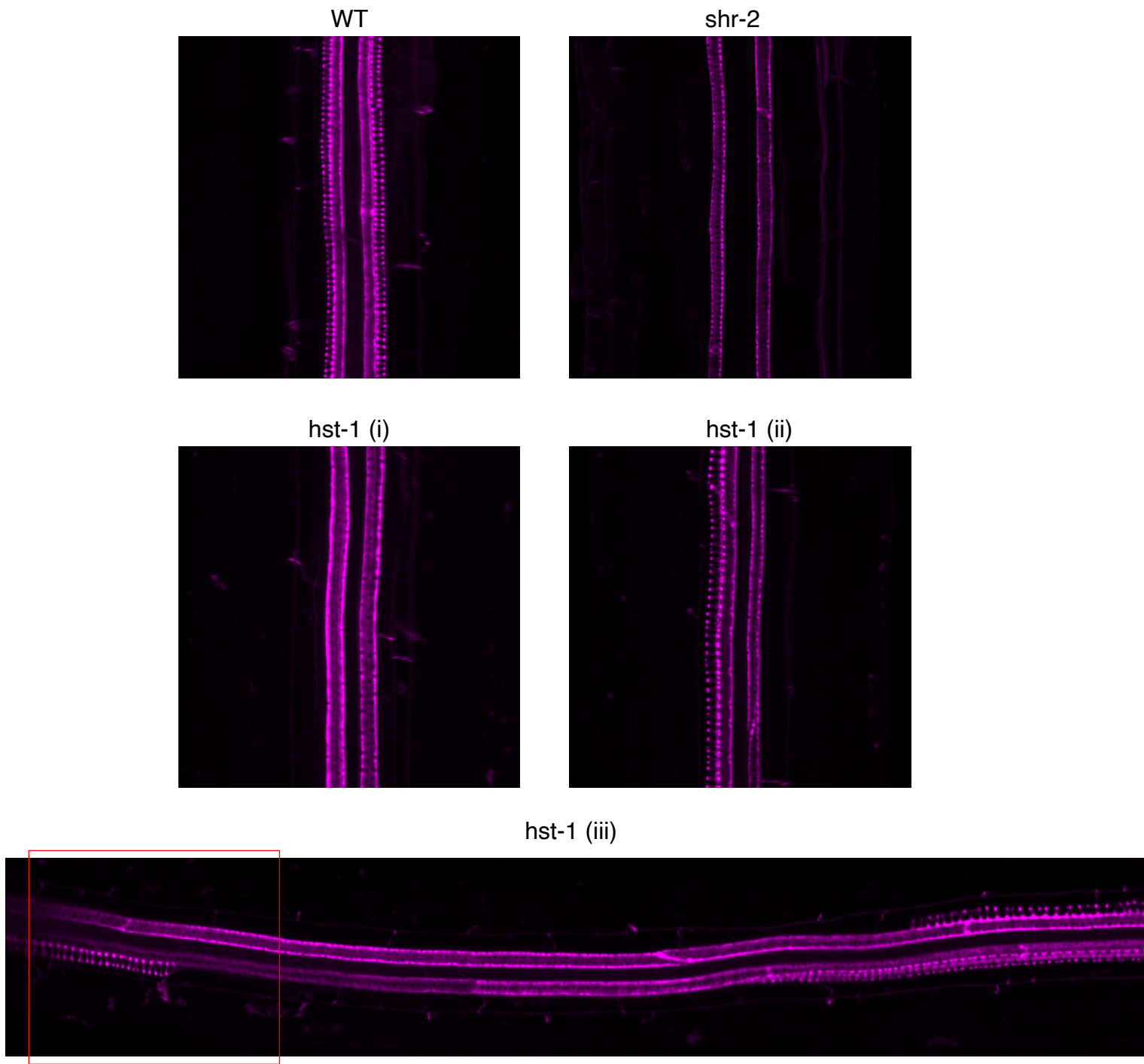

Figure 7F

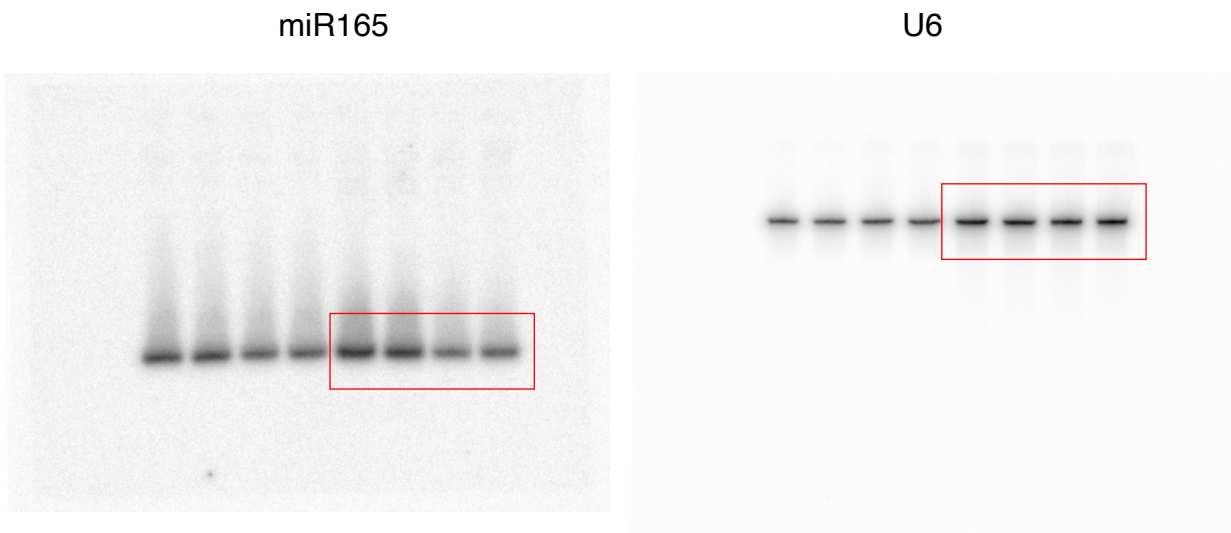

Supplement: Supplementary file 10 — Source Data for Figure 7 [file EMBJ-40-e107455-s009.zip › Raw_data_Fig_7.pdf]
