# Supplementary material for: HASTY, the Arabidopsis EXPORTIN5 ortholog, regulates cell‐to‐cell and vascular microRNA movement
Source: EMBO J. 2021 Jun 21;40(15):e107455. doi: 10.15252/embj.2020107455 (PMC8327949; doi:10.15252/embj.2020107455)

Figure 3A

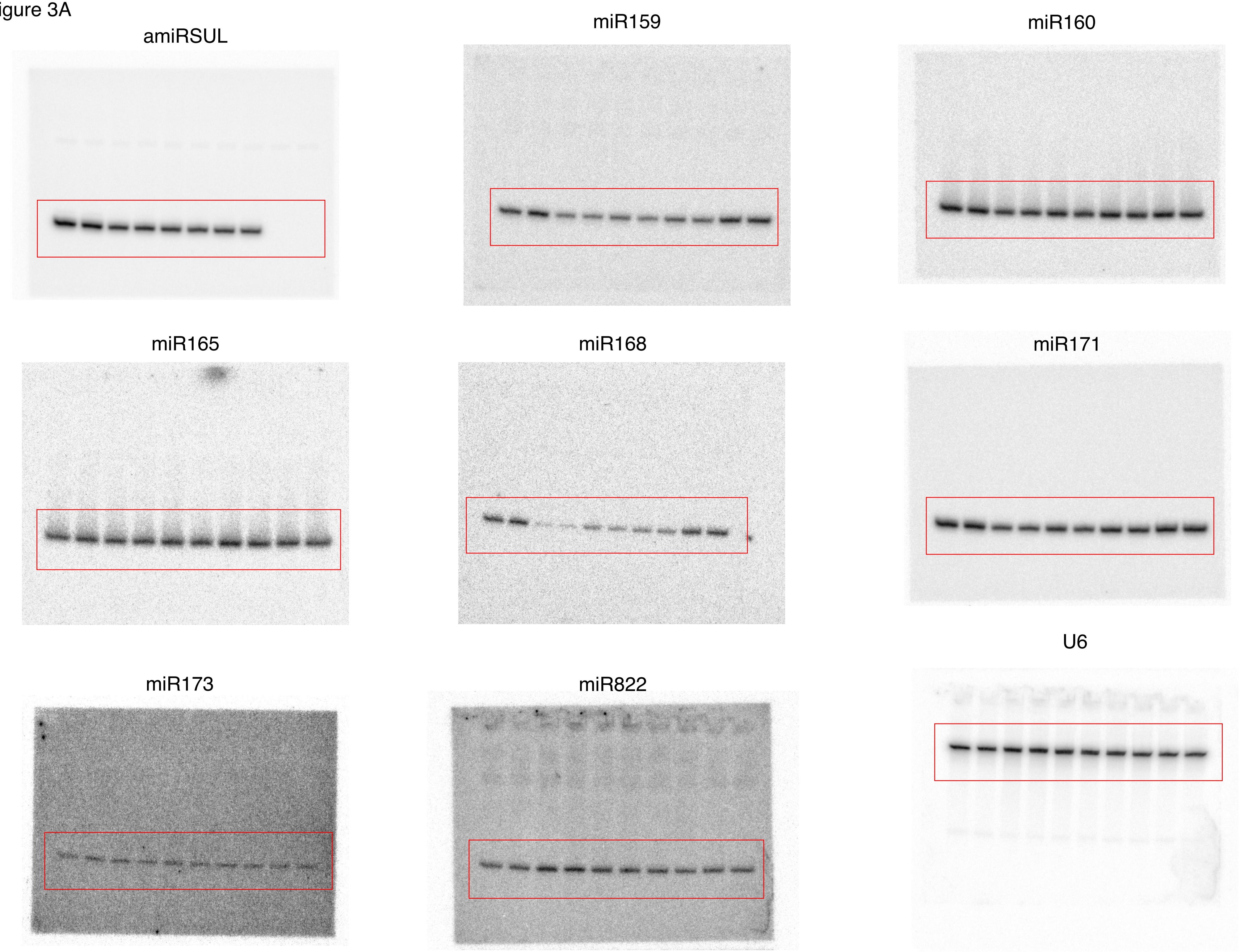
amiRSUL
miR159
miR160
miR165
miR168
miR171
miR173
miR822
U6

Figure 3B

Hom +/+ | Hemi +/-

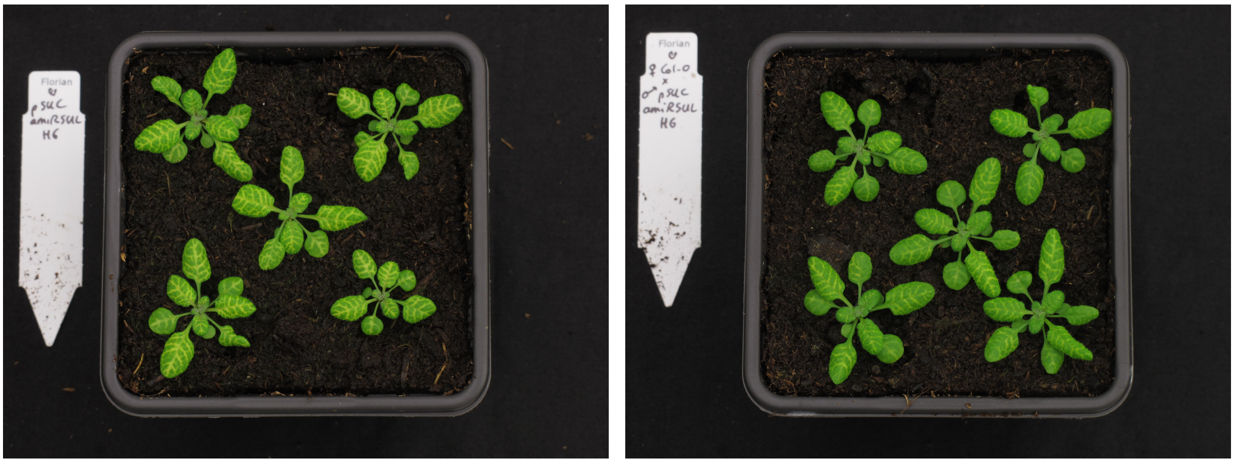

Figure 3C

amiRSUL | miR159 + U6

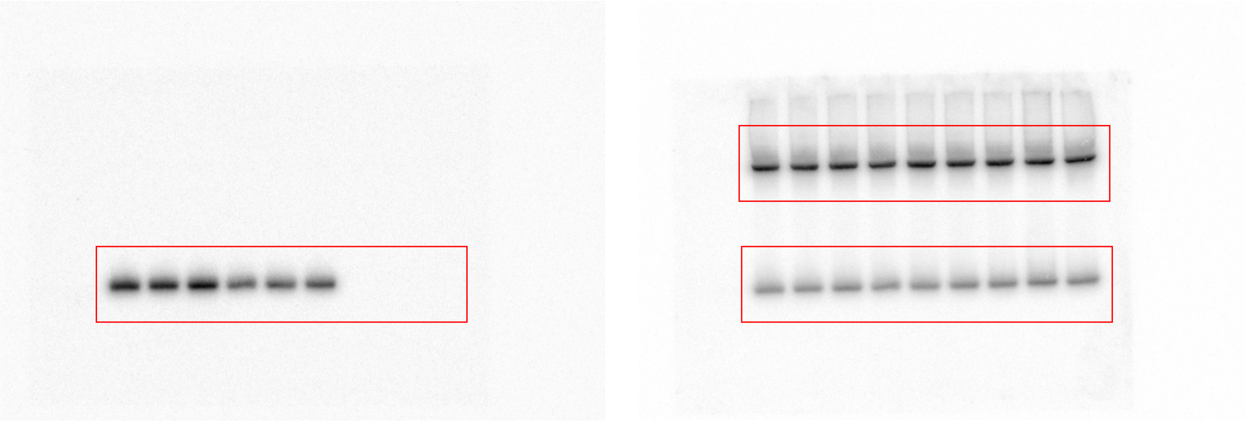

Figure 3D

amiRSUL

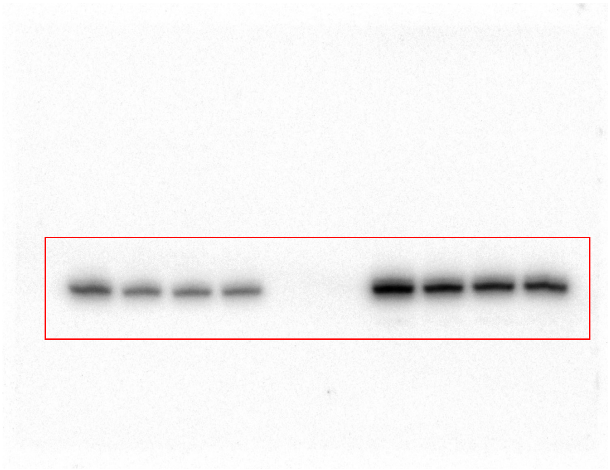

miR165

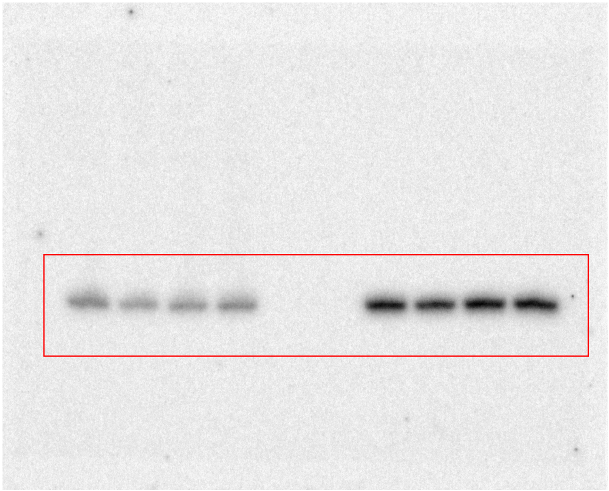

U6

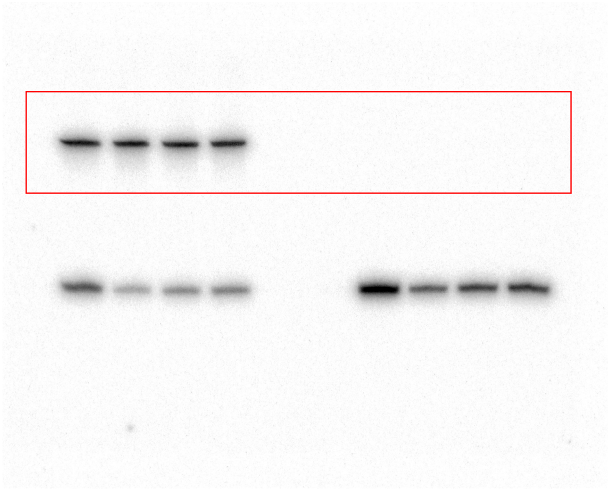

AGO1

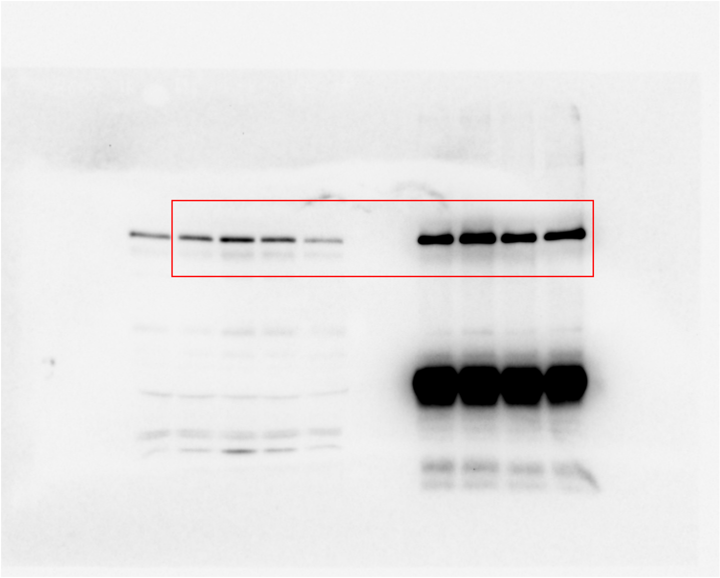

Coom

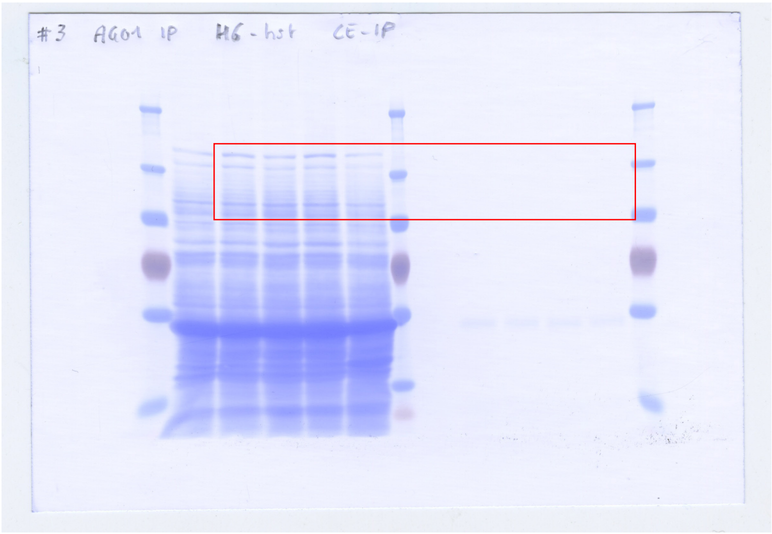

Figure 3E

amiRSUL

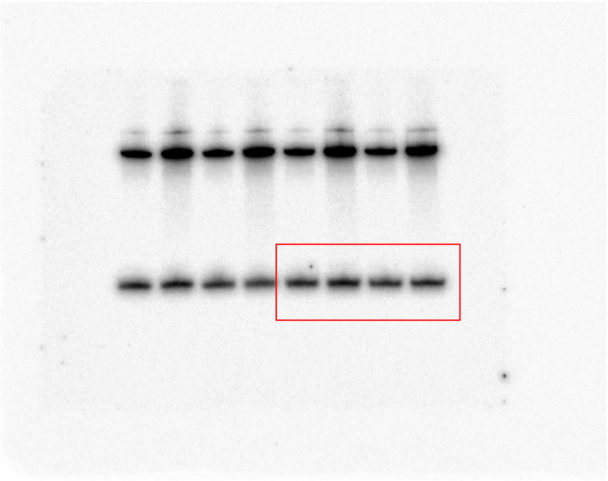

U6

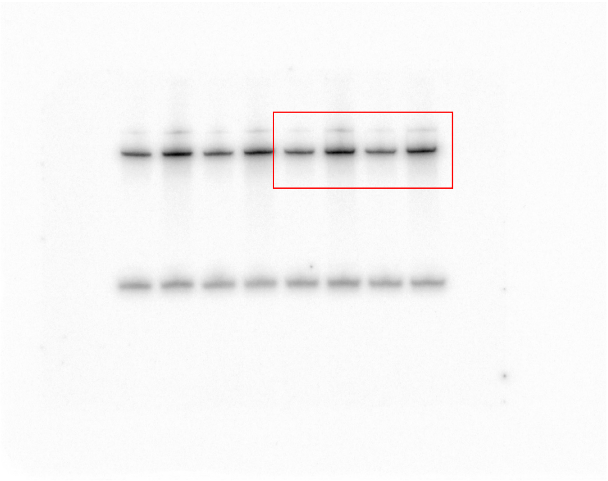

Supplement: Supplementary file 6 — Source Data for Figure 3 [file EMBJ-40-e107455-s003.zip › Raw_data_Fig_3.pdf]
