# Supplementary material for: HASTY, the Arabidopsis EXPORTIN5 ortholog, regulates cell‐to‐cell and vascular microRNA movement
Source: EMBO J. 2021 Jun 21;40(15):e107455. doi: 10.15252/embj.2020107455 (PMC8327949; doi:10.15252/embj.2020107455)

Appendix Figure S1C

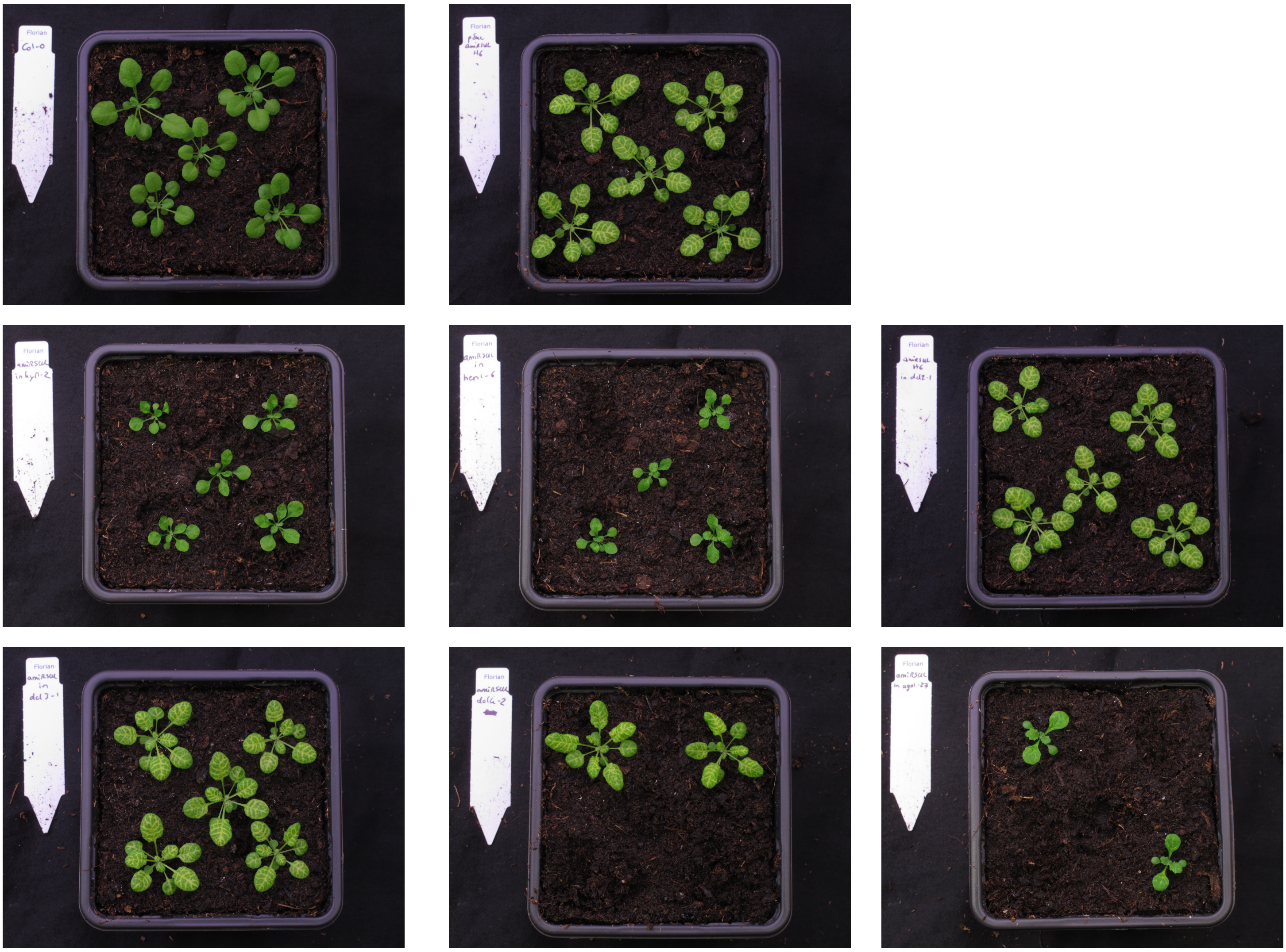

Supplement: Supplementary file 3 — Source Data for Appendix [file EMBJ-40-e107455-s006.zip › Appendix Figure S1 Source Data.zip!/Raw_data_Fig_S1.pdf]
